# Prognostic value of intratumoral *Fusobacterium nucleatum* and association with immune-related gene expression in oral squamous cell carcinoma patients

**DOI:** 10.1038/s41598-021-86816-9

**Published:** 2021-04-12

**Authors:** Cindy Neuzillet, Manon Marchais, Sophie Vacher, Marc Hilmi, Anne Schnitzler, Didier Meseure, Renaud Leclere, Charlotte Lecerf, Coraline Dubot, Emmanuelle Jeannot, Jerzy Klijanienko, Odette Mariani, Valentin Calugaru, Caroline Hoffmann, Maria Lesnik, Nathalie Badois, Edith Borcoman, Eliane Piaggio, Maud Kamal, Christophe Le Tourneau, Ivan Bieche

**Affiliations:** 1 Medical Oncology Department, Institut Curie, Versailles Saint-Quentin University, 35 rue Dailly, 92210 Saint-Cloud, France; 2 Department of Genetics, Institut Curie, PSL Research University, Paris, France; 3 Department of Pathology, Institut Curie, PSL Research University, Paris, France; 4 Department of Drug Development and Innovation (D3i), Institut Curie, Saint-Cloud, Paris, France; 5 Department of Radiotherapy, Institut Curie, PSL Research University, Paris, France; 6 Department of Surgery, Institut Curie, PSL Research University, Paris, France; 7 INSERM U932 Research Unit, Institut Curie, PSL Research University, Paris, France; 8 INSERM U900 Research Unit, Institut Curie, Saint-Cloud, France; 9 Paris-Saclay University, Paris, France; 10 INSERM U1016, Faculty of Pharmaceutical and Biological Sciences, Paris Descartes University, Paris, France

**Keywords:** Cancer microenvironment, Head and neck cancer, Oral cancer, Tumour biomarkers, Tumour immunology

## Abstract

Changes in the oral microbiome, particularly *Fusobacterium nucleatum*, are associated with oral squamous cell carcinoma (OSCC). *F. nucleatum* has been reported to modulate local immunity in cancers. We aimed to assess the association between intratumoral *F. nucleatum* and clinico-pathological features, relapse, and overall survival (OS) in two independent cohorts of patients with OSCC, and to explore the interplay with immune-related genes. We retrospectively analyzed tissue samples from a first cohort of 122 patients with head and neck squamous cell carcinoma, including 61 OSCC (cohort #1), and a second cohort of 90 additional OSCC (cohort #2). We then performed a sensitivity analysis on the merged cohort of OSCC patients (N = 151). *F. nucleatum* 16S rRNA gene sequences were quantified using real-time quantitative PCR. The presence of gram-negative bacteria and macrophages was confirmed by LPS and CD163 immunostainings, respectively. *F. nucleatum* positivity was associated with older age, less alcohol and combined alcohol plus tobacco consumption, and less frequent lymph node invasion. There was a trend for a lower recurrence rate in *F. nucleatum*-positive cases, with less metastatic relapses compared to *F. nucleatum*-negative tumors, and significantly longer OS, relapse-free and metastasis-free survival. *F. nucleatum* status was independently associated with OS in multivariate analysis. Immune-related gene and immunohistochemistry analyses showed that gram-negative bacteria load inversely correlated with M2 macrophages. *F. nucleatum*-associated OSCC has a specific immune microenvironment, is more frequent in older, non-drinking patients, and associated with a favorable prognosis.

## Introduction

Head and neck cancers encompass a variety of tumors originating from the oral cavity, hypopharynx, oropharynx, nasopharynx, or larynx^1^. It is the sixth most common malignancy worldwide, accounting for approximately 5% of all cancer cases and of all cancer deaths^1, 2^. Squamous cell carcinoma is the most frequent histological type, representing more than 90% of these tumors^3^. Classical risk factors for head and neck squamous cell carcinoma (HNSCC) include tobacco and alcohol consumption, as well as human papillomavirus (HPV) infection that has been demonstrated to have a favorable prognostic impact and better response to treatments^4, 5^. Smoking-related HNSCCs demonstrate near universal loss-of-function *TP53* mutations, which are associated with shorter survival and resistance to radiotherapy and chemotherapy^6^. No relevant biomarkers for tailored therapeutic strategies have been identified in HNSCC to date^1^.

Oral squamous cell carcinoma (OSCC) is the most common subset of HNSCC^2^. Nearly two-third of cases are attributed to tobacco smoking and alcohol consumption, and unlike cancers of the oropharynx, only a small fraction of OSCC cases (approximately 4%) are related to HPV infection (mostly, HPV16)^4, 7^.

The human body is inhabited by over 100 trillion microbial cells living in symbiosis with their host^8^. The term microbiome stands for “the collective genomes and gene products of all microbes residing within an organism”^8^. Most of these bacteria are located in the gut. Individual bacteria and shifts in microbiome composition are associated with human disease, including cancer^9, 10^. Hence, the oral bacterial flora plays an essential role in maintaining a healthy oral physiological environment and changes in the oral microbiome have been associated with cancer^11^. Recently, it has been demonstrated that bacteria are also found within tumoral tissue, so called “intratumoral” microbiota^12^. Periodontal pathogens including *Fusobacterium nucleatum* (*F. nucleatum*) are a risk factor for OSCC, independent from tobacco, alcohol, and HPV^11, 13^, and their abundance in saliva samples increases with tumor progression^14, 15^. Little is known about the association between intratumoral *F. nucleatum* and HNSCC tumor biology^16, 17^. Moreover, *F. nucleatum* has been reported to modulate local immunity in other cancers (mainly, colorectal cancers), particularly macrophages and T regulatory cells (Tregs), via Toll like receptor (TLR) 2–4 signaling^18–20^.

In this study, we assessed the association between intratumoral *F. nucleatum* and clinico-pathological features, relapse, and overall survival (OS) in two independent cohorts of patients with OSCC, and explored the interplay between *F. nucleatum* and immune-related genes.

## Patients and methods

### Patients and samples

We retrieved samples from patients with HNSCC who underwent upfront surgery at the Curie Institute between 1990 and 2006. We selected patients with complete clinical, histological, and biological data and long-term follow-up.

**Figure 1:**
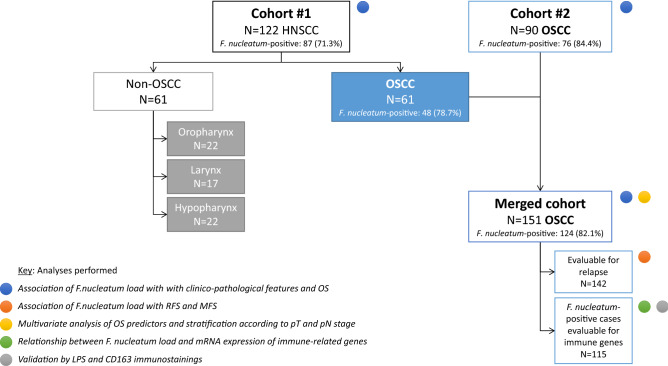
Patient flow-chart.

We used samples from two independent cohorts: the cohort #1 consisted of 122 patients with HNSCC from various primary sites: oral cavity (OSCC; n = 61), oropharynx (n = 22), hypopharynx (n = 17), and larynx (n = 22). The cohort #2 consisted of 90 OSCC patients. Finally, we grouped the OSCC patients from the first cohort with the second cohort for analysis (merged cohort). The flow chart of patients is displayed in Fig. 1.

The study was conducted in accordance with the ethics principles of the Declaration of Helsinki and GDPR regulations. In accordance with the French regulations, informed consent was obtained from all subjects. This study was approved by the French Ethical Committee (Agreement number D-750602, France) and the ethics committee of the Institut Curie (Agreement number C75-05-18).

### Genomic DNA and total RNA extractions

Tumor samples were frozen in liquid nitrogen in a cryotube immediately after surgery and stored at − 80 °C, under temperature control.

A tumor fragment of 5–40 mg and 10–50 mg was used for DNA and RNA extraction, respectively. Tumoral cellularity was evaluated on a 4–5 µm cryosection and samples with less than 50% of tumoral cells were excluded from the study. Nucleic acid extraction was performed on macrodissected tumoral zones, according to the following protocols. Total genomic DNA was extracted from with phenol–chloroform after proteinase K digestion, followed by the precipitation of nucleic acids in ethanol. Total RNA was extracted with miRNeasy Mini kit Qiagen following supplier’s recommendations. The quality of RNA was verified by migration on agarose gel. Nucleic acids were quantified using Nanodrop spectrophotometer ND-1000 (ThermoScientific, Wilmington, DE, USA).

In order to rule out external contaminations for *F. nucleatum* analysis, we included negative controls (buffers/reagents without tumor samples) and the samples were manipulated under a hood and with mask and gloves.

### TP53 and PIK3CA mutation status

Information about *TP53* mutation status was extracted for the results of a Next Generation Sequencing (NGS) panel and *PIK3CA* mutation status was determined by High Resolution Melting (HRM) analysis in all samples (cohort #1 and cohort #2).

### *Fusobacterium nucleatum* status analysis by real-time quantitative PCR

Quantitative values were obtained from the cycle number (Cycle Threshold, Ct value) at which the increase in the fluorescence signal associated with exponential growth of PCR products started to be detected. Detection is performed by the laser detector of the ABI Prism 7900HT Sequence Detection System (Applied Biosystems, ThermoFisher Scientific, Waltham, MA), using PE Biosystems analysis software according to the manufacturer’s manuals.

The precise amount of genomic DNA (based on optical density) and its quality (i.e. lack of extensive degradation) are both difficult to assess. We therefore normalized *F. nucleatum* levels on the basis of *JUN* contents, used as human diploid control. Results, expressed as N-fold differences in *F. nucleatum* 16S rRNA gene copy number and termed “Nfn,” were determined as Nfn = 2^ΔCtsample^, where the ΔCt value of the sample is determined by subtracting the average Ct value of the *F. nucleatum* 16S rRNA gene from the average Ct value of the diploid control gene. *F. nucleatum* Ct values lower than 32 were considered as *F. nucleatum*-positive and *F. nucleatum* Ct values higher than 32 were considered as *F. nucleatum*-negative. For the *F. nucleatum*-positive tumors, the Nfn values were normalized such that a Ct value of 32 was equal to 1, i.e. the smallest accurately quantifiable amount of *F. nucleatum* 16S rRNA gene copy number.

Primers for *F. nucleatum* (targeting the 16S rRNA gene DNA sequence, Upper 5′-AGC GTT TGA CAT CTT AGG AAT GA-3′ and Lower 5′-GCC ATG CAC CAC CTG TCT T-3′) and *JUN* (Genbank accession number NM_002228, Upper 5′-CAC GGC CAA CAT GCT CAG G-3′ and Lower 5′-GCA TGA GTT GGC ACC CAC TGT-3′) were designed with the assistance of Oligo 6.0 computer program (National Biosciences, Plymouth, MN). The primer pair for *F. nucleatum* was selected to be unique relative to the sequences of closely related family member 16S rRNA genes of the *Fusobacterium* species. Agarose gel electrophoresis and DNA sequencing were used to verify the specificity of the *F. nucleatum* PCR amplicon.

Real-time quantitative PCR was performed using the Power SYBR Green master mix (ThermoFisher Scientific, Waltham, MA). The thermal cycling conditions were an initial denaturation step at 95 °C for 10 min, followed by 50 cycles at 95 °C for 15 s and 65 °C/60 °C for 1 min for *JUN*/*F. nucleatum* respectively, and then the melt curve steps. The amplifications specificity was confirmed by melting curve analysis.

### Immune-related gene expression analysis by real-time quantitative reverse transcription PCR

The immune-related genes were selected in collaboration with immunologists from our group, consistent with our previous publications^21, 22^.

Immune-related gene expression levels were normalized on the basis of *TBP* contents (Genbank accession number NM_003194)^23^. We chose *TBP*, which encodes the TATA box binding protein, as endogenous control because the prevalence of its transcripts is moderate, and because there are no known TBP retropseudogenes (retropseudogenes lead to co-amplification of contaminating genomic DNA and thus interfere with RT‐PCR, despite the use of primers in separate exons)^23^. We had previously selected and used the same gene as endogenous control in different cancer types including HNSCC^21, 24^.

Results, expressed as N-fold differences in target gene expression and termed “Ntarget,” were determined as Ntarget = 2^ΔCtsample^, where the ΔCt value of the sample is determined by subtracting the average Ct value of the target gene from the average Ct value of the control gene.

Primers for immune-related genes were designed with the assistance of Oligo 6.0 computer program (National Biosciences, Plymouth, MN). We searched the dbEST and nr databases to confirm the total gene specificity of the nucleotide sequences chosen as primers and the absence of single nucleotide polymorphisms^25^. In particular, the primer pairs were selected to be unique relative to the sequences of closely related family member genes or of the corresponding retropseudogenes^25^. Agarose gel electrophoresis was used to verify the specificity of PCR amplicons. The nucleotide sequences of the oligonucleotide hybridization primers and the average Ct value are shown in Suppl. Table 1. Sixty-four genes mainly involved in the immune process were selected, in particular 19 checkpoint T cell and tumor cell genes, 16 chemokine genes and 18 immune cell population genes. For each primer pair we performed no-template control, no-RT control (RT negative) and RT control with genomic DNA assays, which produced negligible signals that were usually greater than 40 in Ct value, suggesting that primer-dimer formation and genomic DNA contamination effects were negligible.

RNA was reverse transcribed in a final volume of 20 µL containing 1X RT buffer (50 mM Tris–HCl (pH 8.3); 75 mM KCl; 3 mM MgCl2), 10 mM DTT, 100 units of SuperScript II Reverse Transcriptase with reduced RNase H activity (Life Technologies, Carlsbad, CA), 0.5 mM each dNTP (GE Healthcare, Chicago, IL), 0.15 µg/µL random primers (Life Technologies, Carlsbad, CA), 20 units of RNasin Ribonuclease Inhibitor (Promega, Madison, WI), and 1 µg of total RNA. The thermal cycling conditions were hybridization of random primers at 25 °C for 10 min, cDNA synthesis at 42 °C for 30 min, and reverse transcriptase inactivation at 99 °C for 5 min and cooling at 4 °C.

All of the real-time quantitative PCR reactions were performed using the Power SYBR Green master mix (ThermoFisher Scientific, Waltham, MA) using an ABI Prism 7900HT Sequence Detection System (Applied Biosystems, ThermoFisher Scientific, Waltham, MA). The thermal cycling conditions were an initial denaturation step at 95 °C for 10 min, followed by 50 cycles at 95 °C for 15 s and 65 °C for 1 min, and then the melt curve steps. The amplifications specificity was confirmed by melting curve analysis.

### Immunohistochemistry

Immunohistochemistry assay was performed using CD163 (Novocastra Leica, ref.: CD163-L-CE, monoclonal Mouse, clone 10D6, pH9 1/1000, 30 m) and LPS (MyBioSource, ref.: MBS2111237, monoclonal Mouse, clone C8, pH6, 1/500, 60 m) antibodies. Paraffin-embedded tissue blocks, obtained at the time of the initial diagnosis, were retrieved from the archives of the Department of Diagnostic and Theranostic Medicine, Curie Institute. Sections of 3 µm in thickness were cut with a microtome from the paraffin-embedded tissue blocks of OSCCs. Tissue sections were deparaffinized and rehydrated through a series of xylene and ethanol washes. Briefly, key figures included: (1) antigen retrieval in 0.1 mol/L citrate buffer, pH 6 (BioCare, Pacheco, CA, USA) in a pressure cooker (4 min); (2) blocking of endogenous peroxidase activity by immersing sections in 3% hydrogen peroxide in methanol for 15 min and subsequently rinsing them in water and PBS; (3) incubation with primary antibodies against the targeted antigen; (4) immunodetection with a biotin-conjugated secondary antibody formulation that recognizes rabbit and mouse immunoglobulins, followed by peroxidase-labeled streptavidin and linking with a rabbit biotinylated antibody against mouse immunoglobulin G (DAKO SA) and (5) chromogenic revelation with DAB and counterstaining with Mayer’s hematoxylin^26^. All immunostainings were processed using a Leica BOND RX research automated immunostaining device. A semi quantitative score (intensity × frequency) was used for interpretation of immunostaining with anti-LPS antibody (0 = negative staining, 1 = weak staining, 2 = moderate staining and 3 = strong staining). Semi-quantitative evaluation of the tumoral and non-tumoral cells was performed (0: absence of cells, +: < 5%, ++: 5 to 25%, +++: > 25%). Percentage of macrophage immunostaining with anti-CD163 antibody was also evaluated semi-quantitatively using the following score Histologic Score (HS): HS 0: absence of positive cells; HS 1 (+): 1 < HS < 25%; HS 2 (++): 26 < HS < 50%; HS 3 (+++): HS > 50%.

### Statistical methods

Relationships between *F. nucleatum* and clinical, biological, and pathological parameters were assessed by using the Chi-square, Chi-square with Yates correction or Fisher tests, as appropriate. Spearman rank correlation non-parametric test was used to determine relationships between *F. nucleatum* and immune-related genes levels. Bonferroni correction was applied to adjust for multiple tests. Differences were considered significant at confidence levels greater than 95% (*p* < 0.05).

Survival endpoints were defined according to the DATECAN consensus^27^. OS was determined from the time of initial diagnosis to the time of death, regardless of the cause of death. Relapse-free survival (RFS) was determined from the time of initial diagnosis to the time of relapse (locoregional and/or metastatic) or death, whichever occurred first, regardless of the cause of death. Metastasis-free survival (MFS) was determined from the time of initial diagnosis to the time of metastatic relapse or death, whichever occurred first, regardless of the cause of death. In the absence of event, patients were censored at the date of last follow-up. Survival distributions were estimated by the Kaplan–Meier method, and the significance of differences between survival rates were ascertained with the log-rank test. The multivariate Cox proportional hazards regression model was used to assess the prognostic significance of *F. nucleatum* and clinical markers on OS; parameters with *p* values < 0.05 in univariate analysis were entered into the final multivariable Cox regression model, after considering redundancy between variables. The results are presented as hazard ratios (HR) and 95% confidence intervals (95%CIs).

## Results

### Association of *Fusobacterium nucleatum* with clinico-pathological features and overall survival (OS) in the cohort #1

The first cohort (cohort #1) comprised 122 patients with untreated HNSCC from various primary sites, with a majority of tumors arising from the oral cavity (OSCC, n = 61, 50%). Patient characteristics are listed in Suppl. Table 2. Median age was 57 years (range 22–78). Most patients were male (76.2%), with alcohol (64.8%) or tobacco (71.3%) consumption or both (59.1%). Nine patients (7.4%) were HPV-positive. Pathological staging showed a high proportion of UICC stage IV tumors (50.8%). Seventy-two (59%) harbored a mutation in *TP53* gene and 15 (12.3%) in *PIK3CA* gene.

Among the 122 HNSCC samples tested, 35 (28.7%) were scored *F. nucleatum*-negative and 87 (71.3%) were scored *F. nucleatum*-positive. Among the 87 *F. nucleatum*-positive tumors, major differences of Nfn values (determined as described in “Patients and Methods”) were observed, ranging from 1.0 to 1613 (Suppl. Fig. 1).

Association of patient characteristics with *F. nucleatum* status is displayed in Suppl. Table 2. *F. nucleatum* positivity was associated with an enrichment in female (28.7% vs. 11.4%, *p* = 0.042), non-drinking patients (42.4% vs. 13.6%, *p* = 0.014), with more tumors from the oral cavity and oropharynx (55.2% vs. 37.1%, and 21.8% vs. 8.6%, respectively) and less tumors from the hypopharynx (5.7% vs. 34.3%, *p* = 0.0003).

Alcohol (*p* = 0.019) and tobacco (*p* = 0.017) consumption, pT (*p* = 0.021) and pN (*p* = 0.045) and UICC (*p* = 0.013) stage, absence of HPV infection (*p* = 0.0061), presence of *TP53* mutation (*p* = 0.0007), and distant (metastatic) relapse (*p* = 0.0085) were significantly associated with shorter OS in univariate analysis (Suppl. Table 3).

**Figure 2:**
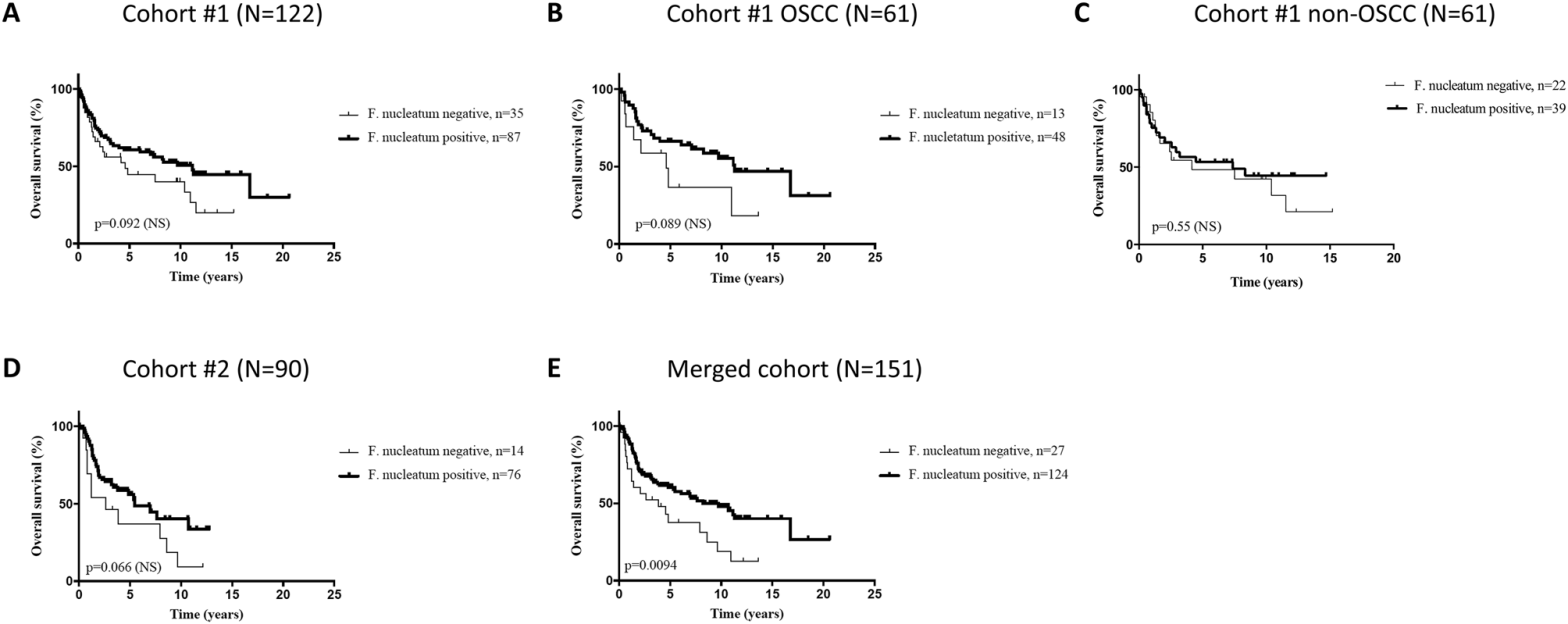
Overall survival (OS) curves according to *F. nucleatum* status (**A**) in the overall head and neck squamous cell carcinoma (HNSCC) patient population (N = 122), (**B**) in the oral cavity tumor (OSCC) subgroup (N = 61) of cohort #1, (**C**) in the non-OSCC subgroup (N = 61) of cohort #1, (**D**) in cohort #2 (N = 90), and (**E**) in the merged cohort (N = 151).

In this overall HNSCC population, there was a non-significant trend toward longer OS in patients with *F. nucleatum*-positive tumors (HR: 0.64, *p* = 0.092) (Fig. 2A). We analyzed the association of *F. nucleatum* status with OS according to tumor primary site (Fig. 2B,C and Suppl. Fig. 2). We observed that this trend was mainly driven by the oral cavity subgroup (HR: 0.51, *p* = 0.089) (Fig. 2B), while there was no significant association with OS in patients with HNSCC tumors from other primary sites (Fig. 2C and Suppl. Fig. 2).

Therefore, we decided to focus on the subset of patients with oral cavity tumors for the next steps. In this subgroup of 61 OSCC patients from cohort #1, *F. nucleatum* positivity (78.7%) was associated with lower pN stage (*p* = 0.043) and a non-significant trend for more non-drinkers (*p* = 0.092) compared to *F. nucleatum*-negative tumors (Suppl. Table 4). Among the clinical, biological and pathological parameters listed above, *TP53* mutation (HR: 2.76, *p* = 0.0093) and UICC stage (HR: 2.79, *p* = 0.043 for stage II and HR: 2.70, *p* = 0.049 for stage IV) were significantly associated with OS (Table 1).

**Table 1:** Clinical, biological and pathological characteristics of the oral cavity (OSCC) patients sets: 61 patients from cohort #1, 90 patients of cohort #2 and 151 patients of the merged cohort, in relation with overall survival (OS).

	Cohort #1 OSCC (n = 61)					Cohort #2 (n = 90)					Merged cohort (n = 151)				
	Patients (%)	Death (%)	HR^a^	95% CI^b^	OS^c^	Patients (%)	Death (%)	HR^a^	95% CI^b^	OS^c^	Patients (%)	Death (%)	HR^a^	95% CI^b^	OS^c^
**Total**	61 (100)	31 (50.8)				90 (100)	46 (51.1)				151 (100)	77 (51)			
**Age**															
< 56	24 (39.3)	11 (45.8)	1		0.73 (NS)	21 (23.3)	8 (38.1)	1		0.25 (NS)	45 (29.8)	19 (42.2)	1		0.20 (NS)
≥ 56	37 (60.7)	20 (54.1)	1.14	0.55–2.34		69 (76.7)	38 (55.1)	1.55	0.79–3.03		106 (70.2)	58 (54.7)	1.40	0.86–2.26	
**Sex**															
Female	24 (39.3)	10 (41.7)	1		0.44 (NS)	34 (37.8)	19 (55.9)	1		0.78 (NS)	58 (38.4)	29 (50)	1		0.72 (NS)
Male	37 (60.7)	21 (56.8)	1.34	0.65–2.76		56 (62.2)	27 (48.2)	0.92	0.51–1.67		93 (61.6)	48 (51.6)	1.09	0.69–1.72	
**Alcohol**^**d**^															
No	22 (45.8)	8 (36.4)	1		0.20 (NS)	57 (70.4)	32 (56.1)	1		0.53 (NS)	79 (61.2)	40 (50.6)	1		0.99 (NS)
Yes	26 (54.2)	14 (53.8)	1.74	0.75–4.01		24 (29.6)	10 (41.7)	0.80	0.41–1.57		50 (38.8)	24 (48)	1.00	0.60–1.66	
**Tobacco**^**e**^															
No	20 (38.5)	6 (30)	1		0.077 (NS)	42 (48.8)	22 (52.4)	1		0.89 (NS)	62 (44.9)	28 (45.2)	1		0.36 (NS)
Yes	32 (61.5)	19 (59.4)	2.23	1.01–4.94		44 (51.2)	22 (50)	0.96	0.53–1.73		76 (55.1)	41 (53.9)	1.25	0.78–2.01	
**Alcohol and tobacco**^**d**^															
No	25 (52.1)	9 (36.0)	1		0.20 (NS)	59 (72.8)	33 (55.9)	1		0.48 (NS)	84 (65.1)	42 (50.0)	1		0.95 (NS)
Yes	23 (47.9)	13 (56.5)	1.72	0.74–3.97		22 (27.2)	9 (40.9)	0.77	0.39–1.53		45 (34.9)	22 (48.9)	0.98	0.59–1.65	
**pT**															
1	14 (23.0)	6 (42.9)	1		0.11 (NS)	7 (7.8)	1 (14.3)	1		0.11 (NS)	21 (13.9)	7 (33.3)	1		0.015*
2	14 (23.0)	7 (50.0)	1.70	0.56–5.14	0.33 (NS)	24 (26.7)	10 (41.7)	3.23	0.82–12.8	0.24 (NS)	38 (25.2)	17 (44.7)	1.93	0.86–4.31	0.13 (NS)
3	21 (34.4)	13 (61.9)	2.43	0.99–5.99	0.057 (NS)	37 (41.1)	22 (59.5)	5.36	1.91–15.0	0.065 (NS)	58 (38.4)	35 (60.3)	3.20	1.72–5.94	0.0024**
4	12 (19.7)	5 (41.7)	1.14	0.35–3.78	0.82 (NS)	22 (24.4)	13 (59.1)	5.44	1.73–17.2	0.064 (NS)	34 (22.5)	18 (52.9)	2.24	1.02–4.92	0.057 (NS)
**pN**															
0	34 (55.7)	17 (50.0)	1		0.38 (NS)	47 (52.2)	19 (40.4)	1		0.0065**	81 (53.6)	36 (44.4)	1		0.0040**
1	5 (8.2)	2 (40.0)	0.88	0.22–3.57	0.87 (NS)	10 (11.1)	6 (60.0)	2.42	0.71–8.24	0.045*	15 (9.9)	8 (53.3)	1.66	0.66–4.13	0.19 (NS)
2	10 (16.4)	6 (60.0)	1.77	0.59–5.28	0.22 (NS)	13 (14.4)	8 (61.5)	2.05	0.75–5.58	0.081 (NS)	23 (15.2)	14 (60.9)	1.98	0.94–4.18	0.026*
3	12 (19.7)	6 (50.0)	2.10	0.66–6.74	0.098 (NS)	20 (22.2)	13 (65.0)	3.03	1.24–7.42	0.0010**	32 (21.2)	19 (59.4)	2.62	1.29–5.30	0.0003**
**Differentiation**^**f**^															
Verrucous	0 (0)	0 (0)			0.82 (NS)	4 (4.5)	1 (25.0)	0.33	0.10–1.11	0.25 (NS)	4 (2.8)	1 (25.0)	0.37	0.11–1.26	0.30 (NS)
Grade I	43 (76.8)	23 (53.5)	1			63 (70.8)	30 (47.6)	1		0.041*	106 (73.1)	53 (50.0)	1		0.27 (NS)
Grade II	8 (14.3)	4 (50.0)	1.35	0.41–4.40	0.58 (NS)	19 (21.3)	11 (57.9)	1.59	0.73–3.48	0.18 (NS)	27 (18.6)	15 (55.6)	1.54	0.80–2.97	0.13 (NS)
Grade III	5 (8.9)	2 (40.0)	0.81	0.22–3.05	0.78 (NS)	3 (3.4)	3 (100)		0.44–26.2	0.031*	8 (5.5)	5 (62.5)	1.35	0.48–3.82	0.41 (NS)
Grade IV	0 (0)	0 (0)				0 (0)	0 (0)	3.39			0 (0)	0 (0)			
**Margins**^**g**^															
Negative	44 (78.6)	22 (50.0)	1		0.33 (NS)	78 (86.7)	39 (50.0)	1		0.17 (NS)	122 (83.6)	61 (50.0)	1		0.16 (NS)
Positive	12 (21.4)	7 (58.3)	1.51	0.58–3.95		12 (13.3)	7 (58.3)	1.73	0.64–4.67		24 (16.4)	14 (58.3)	1.51	0.77–2.94	
**HPV**															
Negative	60 (98.4)	31 (51.7)	ND		0.34 (NS)	87 (96.7)	44 (50.6)	1		0.89 (NS)	147 (97.4)	75 (51)	1		0.56 (NS)
Positive	1 (1.6)	0 (0)				3 (3.3)	2 (66.7)	0.91	0.23–3.58		4 (2.6)	2 (50)	0.66	0.21–2.12	
**UICC stage**															
Stage I	15 (24.6)	5 (33.3)	1		0.20 (NS)	5 (5.6)	0 (0)			0.024*	20 (13.2)	5 (25)	1		0.0036**
Stage II	19 (31.1)	11 (57.9)	2.79	1.04–7.48	0.043*	18 (20)	6 (33.3)	1		0.16 (NS)	37 (24.5)	17 (45.9)	2.72	1.17–6.30	0.038*
Stage III	9 (14.8)	4 (44.4)	1.80	0.44–7.40	0.37 (NS)	21 (23.3)	12 (57.1)	1.87	0.74–4.72	0.060 (NS)	30 (19.9)	16 (53.3)	3.56	1.51–8.38	0.0071**
Stage IV	18 (29.5)	11 (61.1)	2.70	1.01–7.23	0.049*	46 (51.1)	28 (60.9)	2.44	1.20–4.95	0.030*	64 (42.4)	39 (60.9)	4.32	2.33–8.01	0.0005***
**TP53 mutational status**															
Wild-type	27 (44.3)	8 (29.6)	1		0.0093**	62 (68.9)	35 (56.5)	1		0.23 (NS)	55 (36.4)	19 (34.5)	1		0.0033**
Mutated	34 (55.7)	23 (67.6)	2.76	1.37–5.59		28 (31.1)	11 (39.3)	1.51	0.81–2.80		96 (63.6)	58 (60.4)	2.13	1.35–3.35	
**PIK3CA mutational status**															
Wild-type	54 (88.5)	27 (50)	1		0.49 (NS)	79 (87.8)	38 (48.1)	1		0.31 (NS)	133 (88.1)	65 (48.9)	1		0.17 (NS)
Mutated	7 (11.5)	4 (57.1)	1.44	0.43–4.81		11 (12.2)	8 (72.7)	1.48	0.62–3.54		18 (11.9)	12 (66.7)	1.53	0.74–3.13	
**Relapse**															
No	36 (59.0)	16 (44.4)	1		0.37 (NS)	57 (63.3)	17 (29.8)	1		< 0.0001**	93 (61.6)	33 (35.5)	1		< 0.0001**
Yes	25 (41.0)	15 (60.0)	1.37	0.67–2.81		33 (36.7)	29 (87.9)	5.16	2.64–10.1		58 (38.4)	44 (75.9)	2.75	1.71–4.43	
**Locoregional relapse**	17 (68.0)	9 (52.9)	1		0.23 (NS)	21 (63.6)	18 (85.7)	1		0.083 (NS)	38 (65.5)	27 (71.1)	1		0.017*
**Distant metastasis**	6 (24.0)	4 (66.7)	1.40	0.39–4.98	0.56 (NS)	5 (15.2)	5 (100)	1.72	0.53–5.62	0.27 (NS)	11 (19.0)	9 (81.8)	1.40	0.61–3.21	0.37 (NS)
**Both**	2 (8.0)	2 (100)	3.40	0.29–40.0	0.088 (NS)	7 (21.2)	6 (85.7)	2.70	0.75–9.72	0.025*	9 (15.5)	8 (88.9)	3.0	0.94–9.60	0.0033**

Significant (*p* < 0.05) variables are highlighted in bold.

OS: overall survival; NS: not significant; HPV: human papilloma virus; UICC: Union for International Cancer Control; ND: Undefined.

*p < 0.05.

**p < 0.01.

***p < 0.001.

^a^Hazard ratio (logrank).

^b^95% Confidence interval.

^c^Log-rank test.

^d^Information available for 48, 81 and 129 patients of cohort #1, cohort #2 and merged cohort respectively.

^e^Information available for 52, 86 and 138 patients of cohort #1, cohort #2 and merged cohort respectively.

^f^Information available for 56, 89 and 145 patients of cohort #1, cohort #2 and merged cohort respectively.

^g^Information available for 56, 90 and 146 patients of cohort #1, cohort #2 and merged cohort respectively.

### Association of *Fusobacterium nucleatum* with clinico-pathological features and OS in the cohort #2

To further explore the association of *F. nucleatum* with OS in patients with OSCC, we used an independent cohort composed of 90 patients with HNSCC tumors exclusively from this primary site (cohort #2). Patient characteristics and association with *F. nucleatum* status as displayed in Suppl. Table 5. *F. nucleatum* was detected in 76 (84.4%) tumors. Most OSCC patients were men (62.2%), ≥ 56 years (76.7%), non-drinkers (70.4%) but smokers (51.2%), with HPV-negative (96.7%), advanced (mainly, stage IV: 51.1%) tumors. *F. nucleatum* status was associated with patient age (*p* = 0.0036), pN stage (*p* = 0.028) and tumor grade (*p* = 0.040). Advanced UICC stage (HR: 2.44, *p* = 0.030 for stage IV), high pN stage (HR: 2.42, *p* = 0.045 for pN1, and HR: 3.03, *p* = 0.0010 for pN3), and grade (HR: 3.39, *p* = 0.031 for grade III, and tumor relapse (HR: 5.16, *p* < 0.0001) were associated with poor OS (Table 1). Similarly to the cohort #1, we observed a non-significant trend toward longer OS in the *F. nucleatum*-positive group (HR: 0.54, *p* = 0.066) (Fig. 2D).

### Sensitivity analysis for the association of *Fusobacterium nucleatum* with OS in the merged cohort

In order to increase the study power, we performed a sensitivity analysis after grouping the two cohorts of patients with OSCC tumors (merged cohort, n = 151). Patients characteristics are described in Table 2. As previously observed, patients were mostly men (61.6%), ≥ 56 years (70.2%), non-drinkers (61.2%) but smokers (55.1%), with HPV-negative (97.4%), pN0 (53.6%), UICC stage IV (42.4%) tumors. In the merged cohort, 124 (82.1%) samples were positive for *F. nucleatum* (distribution of Nfn value displayed in Suppl. Figure 1), which was associated with older age (≥ 56 years: 74.2% vs. 51.9%, *p* = 0.021), fewer drinkers (34.9% vs. 60.0%, *p* = 0.034), and less frequent lymph node involvement (more pN0 tumors, 58.1% vs 33.3%, *p* = 0.0016) (Table 2). Positive pN status (*p* = 0.0040) and higher UICC stage (stage ≥ II, *p* = 0.0036), presence of *TP53* mutation (HR: 2.13, *p* = 0.0033), and tumor relapse (HR: 2.75, *p* < 0.0001) were prognostic indicators (Table 1). In the merged cohort, the outcome of the patients with a *F. nucleatum*-positive tumor was significantly better than that of the 27 patients with a *F. nucleatum*-negative tumor in term of OS (HR: 0.51, *p* = 0.0094; 5-year OS 60.5% vs. 37.7%; 10-year OS 47.9% vs. 18.8%) (Fig. 2E).

**Table 2:** Relationship between *F. nucleatum* status and clinical, biological and pathological characteristics of the 151 patients of the merged cohort.

	Patients (%)	Number of patients (%)		*p*-value^a^
		*F. nucleatum* negative	F*. nucleatum* positive	
**Total**	151 (100)	27 (17.9)	124 (82.1)	
**Age**				
< 56	45 (29.8)	13 (48.1)	32 (25.8)	0.021*
≥ 56	106 (70.2)	14 (51.9)	92 (74.2)	
**Sex**				
Female	58 (38.4)	7 (25.9)	51 (41.1)	0.14 (NS)
Male	93 (61.6)	20 (74.1)	73 (58.9)	
**Alcohol**^b^				
No	79 (61.2)	8 (40)	71 (65.1)	0.034*
Yes	50 (38.8)	12 (60)	38 (34.9)	
**Tobacco**^c^				
No	62 (44.9)	7 (28)	55 (48.7)	0.060 (NS)
Yes	76 (55.1)	18 (72)	58 (51.3)	
**Alcohol and tobacco**^b^				
No	84 (65.1)	9 (45.0)	75 (68.8)	0.040*
Yes	45 (34.9)	11 (55.0)	34 (31.2)	
**pT**				
1	21 (13.9)	3 (11.1)	18 (14.5)	0.30 (NS)
2	38 (25.2)	7 (25.9)	31 (25.0)	
3	58 (38.4)	14 (51.9)	44 (35.5)	
4	34 (22.5)	3 (11.1)	31 (25.0)	
**pN**				
0	81 (53.6)	9 (33.3)	72 (58.1)	0.0016**
1	15 (9.9)	0 (0)	15 (12.1)	
2	23 (15.2)	6 (22.2)	17 (13.7)	
3	32 (21.2)	12 (44.4)	20 (16.1)	
**Differentiation**^d^				
Verrucous	4 (2.8)	0 (0)	4 (3.4)	0.062 (NS)
Grade I	106 (73.1)	20 (76.9)	87 (73.1)	
Grade II	27 (18.6)	3 (11.5)	24 (20.2)	
Grade III	8 (5.5)	4 (15.4)	4 (3.4)	
Grade IV	0 (0)	0 (0)	0 (0)	
**Margins**^e^				
Negative or close	122 (83.6)	20 (76.9)	102 (85.0)	0.47 (NS)
Positive	24 (16.4)	6 (23.1)	18 (15.0)	
**HPV**				
Negative	147 (97.4)	25 (92.6)	122 (98.4)	0.15 (NS)
Positive	4 (2.6)	2 (7.4)	2 (1.6)	
**UICC stage**				
Stage I	20 (13.2)	2 (7.4)	18 (14.5)	0.32 (NS)
Stage II	37 (24.5)	4 (14.8)	33 (26.6)	
Stage III	30 (19.9)	6 (22.2)	24 (19.4)	
Stage IV	64 (42.4)	15 (55.6)	49 (39.5)	
**TP53 mutational status**				
Wild-type	55 (36.4)	8 (29.6)	47 (37.9)	0.42 (NS)
Mutated	96 (63.6)	19 (70.4)	77 (62.1)	
**PIK3CA mutational status**				
Wild-type	133 (88.1)	23 (85.2)	110 (88.7)	0.85 (NS)
Mutated	18 (11.9)	4 (14.8)	14 (11.3)	
**Relapse**				
No	93 (61.6)	13 (48.2)	80 (64.5)	0.11 (NS)
Yes	58 (38.4)	14 (51.8)	44 (35.5)	
Locoregional relapse	38 (65.5)	8 (53.3)	31 (70.5)	0.041*
Distant metastasis	11 (19.0)	6 (40.0)	5 (11.4)	
Both	9 (15.5)	1 (6.7)	8 (18.2)	

*HPV* human papilloma virus, *UICC* Union for international cancer control, *NS* Not significant.

*p < 0.05.

**p < 0.01.

***p < 0.001.

^a^Chi-square test, Chi-square test with Yates’ correction or Fisher test if appropriate.

^b^Information available for 129 patients.

^c^Information available for 138 patients.

^d^Information available for 145 patients.

^e^Information available for 146 patients.

### Relapse-free (RFS) and metastasis-free survival (MFS) in the merged cohort

One hundred and fourty two patients were evaluable for disease relapse. There was a non-significant trend for less frequent disease recurrence in patients with *F. nucleatum*-positive tumors (35.5% vs. 51.8%, *p* = 0.11), with more locoregional (70.5% vs. 53.3%, *p* = 0.041) vs metastatic relapse compared to *F. nucleatum*-negative tumors (Table 2).

**Figure 3:**
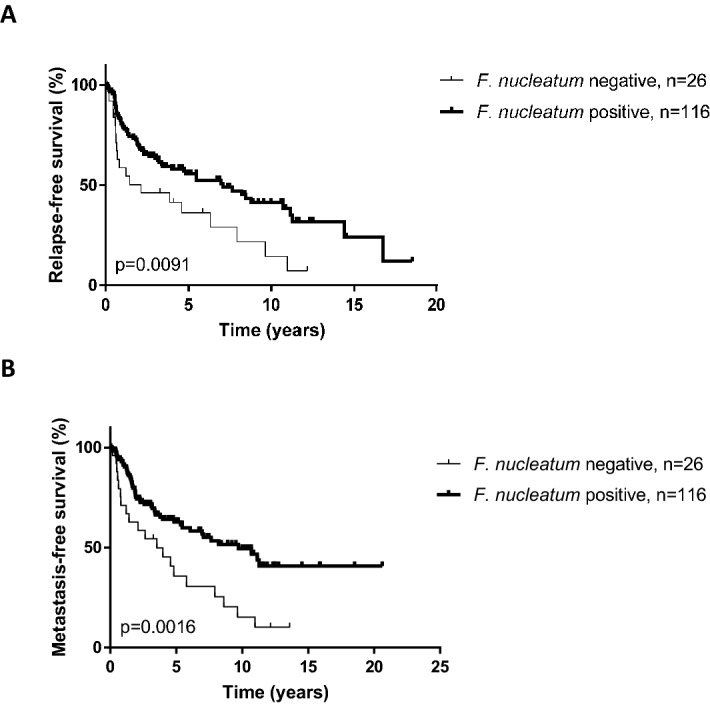
Relapse-free (RFS) (**A**) and metastasis-free survival (MFS) (**B**) curves according to *F. nucleatum* status in the merged cohort (N = 142 evaluable patients).

RFS and MFS curves according to *F. nucleatum* status are displayed in Fig. 3. *F. nucleatum*-positive tumors were associated with significantly longer RFS (median: 7.06 vs. 2.11 months, *p* = 0.0091) and MFS (9.71 vs. 3.54 months, *p* = 0.0016) compared to *F. nucleatum*-negative tumors.

### Multivariate analysis of OS predictors and stratification according to pT and pN stage

Using a Cox proportional hazards model, we also assessed the prognostic value for OS of parameters that were significant in univariate analysis, i.e. pT, pN, UICC stage, *TP53* mutation (Table 1) and *F. nucleatum* status (Fig. 2E). As pT/pN and UICC were redundant variables, the model was built with UICC stage, TP53 and *F. nucleatum*. The prognostic significance of these three parameters was maintained in Cox multivariate regression analysis (Table 3).

**Table 3:** Multivariate Cox analysis of overall survival (OS) for *F. nucleatum* status in the total oral cavity population of 151 patients of the merged cohort.

Characteristics	HR^a^	95% CI^b^	*p* value^c^
***F. nucleatum*** status			
Negative	1		0.028
Positive	0.56	0.33–0.94	
**UICC stage**			
Stage I	1		
Stage II	2.73	1.00–7.45	0.049 ^d^
Stage III	3.10	1.12–8.52	0.029 ^e^
Stage IV	4.19	1.63–10.8	0.0030^f^
**TP53 mutational status**			
Wild-type	1		0.021
Mutated	1.86	1.10–3.14	

^a^Hazard ratio.

^b^95% Confidential interval.

^c^Multivariate cox analysis.

^d^Stages I versus II.

^e^Stages I versus III.

^f^Stages I versus IV.

**Figure 4:**
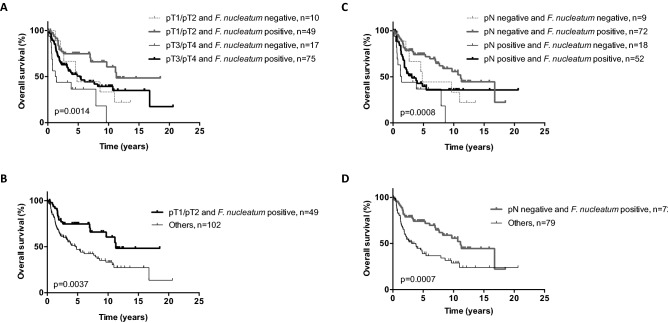
Overall survival (OS) curves according to pT stage and *F. nucleatum* status (**A**,**B**) and according to pN stage and *F. nucleatum* status (**C**,**D**) in the merged cohort (N = 151).

In addition, we assessed the prognostic value of *F. nucleatum* on OS after stratification on pT and pN as main prognostic factors in OSCC. Interestingly, the combinations of pT1/T2 and *F. nucleatum* positivity (n = 49) and pN0 and *F. nucleatum* positivity (n = 72) identify a subgroup of patients with a very favorable survival (Fig. 4).

### Relationship between *Fusobacterium nucleatum* load and mRNA expression of immune-related genes

To explore the underlying mechanisms of the favorable prognosis of *F. nucleatum*-positive tumors, as several studies recently suggested that *F. nucleatum* modulates the local immunity of various cancers (particularly, macrophages and Tregs)^18–20^, we tested possible association between *F. nucleatum* load and expression of various immune-related genes in 115 evaluable *F. nucleatum*-positive samples from the merged cohort (Suppl. Table 6). We observed a significant negative association between *F. nucleatum* load and markers of B lymphocytes (CD20, *p* = 0.027), T helper lymphocytes (CD4, *p* = 0.013), M2 macrophages (CD163, *p* = 0.020), and fibroblasts (PDGFRß, *p* = 0.0067). Toll-like receptor (TLR) 4 (*p* = 0.020) and OX40 ligand (TNFSF4) (*p* = 0.0067) expressions were significantly decreased in tumors with high *F. nucleatum* load. Notably, TNFSF9 receptor (TNFRSF9) expression was decreased (*p* = 0.0067) while the expression of its ligand (TNFSF9) increased with *F. nucleatum* load, along with the pro-inflammatory cytokine IL-1ß (*p* = 0.020). Correlations between *F. nucleatum* load and *TNFSF4*, *TNFSF9*, *IL1B*, and *CD163* expression levels are displayed in Supplementary Fig. 3. There was no association with cell proliferation and APOBEC genes.

### Validation by LPS and CD163 immunostainings

**Figure 5:**
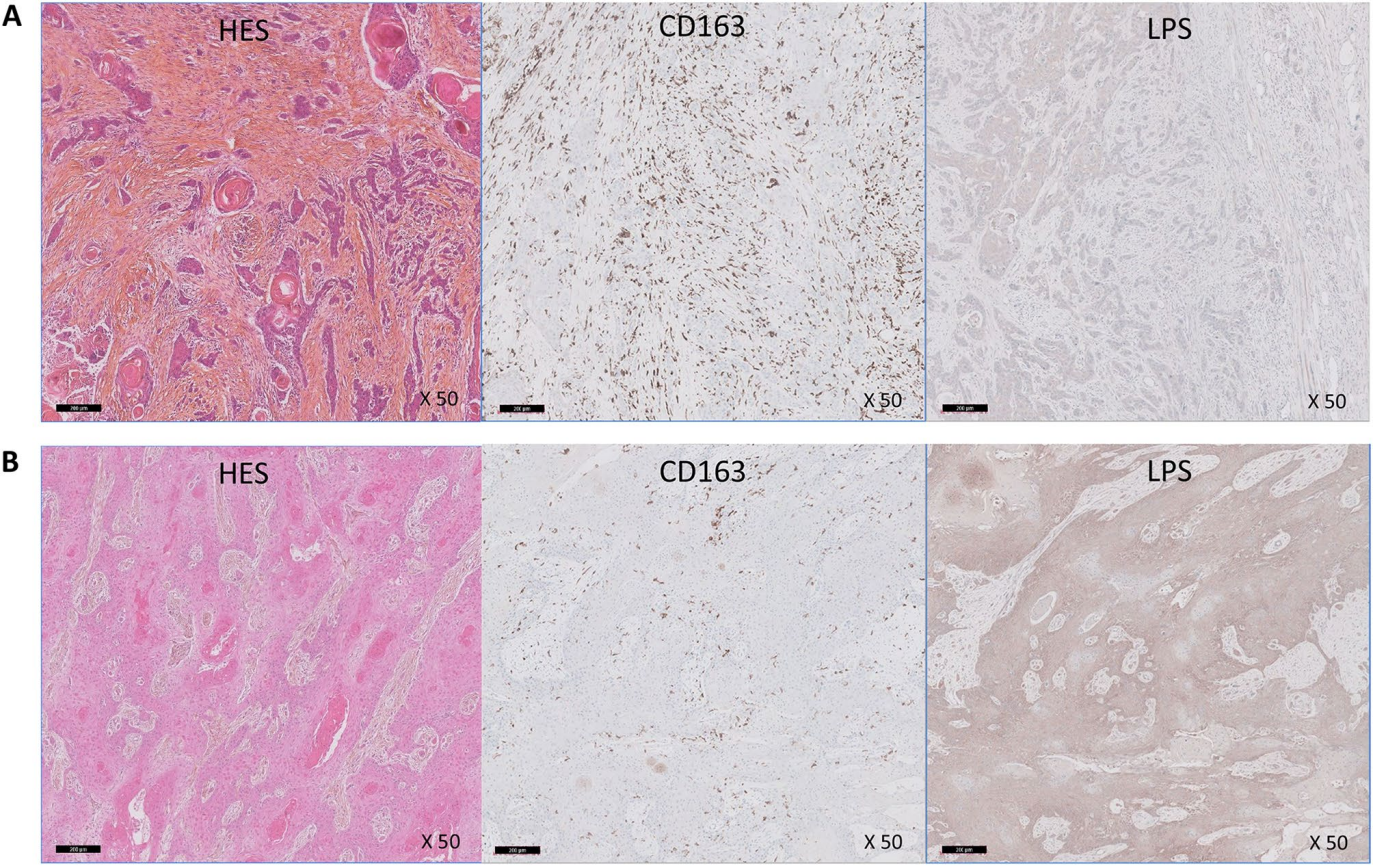
Representative pictures of infiltrating squamous cell carcinomas of the oropharynx: hematoxylin–eosin–safran (HES) stained slides, CD163 and lipopolysaccharide (LPS) immunostainings in a CD163 high/LPS low case (CD163: Histologic Score [HS] 3 (> 50%), LPS: slight expression; *F. nucleatum* negative) (**A**) and in a CD163 low/LPS high case (CD163: HS 1 (< 25%), LPS: strong expression; *F. nucleatum* positive) (**B**) (scale bar = 200 µM; original magnification: ×50).

In order to validate the interplay between bacteria and immune cells, particularly macrophages, we performed immunostainings to detect bacteria using an anti-lipopolysaccharide (LPS) antibody and an anti-CD163 labelling M2 macrophages. We show that, consistent with our PCR results, tumors with high (or low) CD163 and *F. nucleatum* RNA levels display high (or low) CD163 and LPS expressions, respectively, with an inverse correlation between CD163 and LPS expression levels (Fig. 5 and Suppl. Table 7). LPS staining was located mainly in the cytoplasm of tumor and macrophage cells and more rarely in the form of extracellular bacterial vesicles (Suppl. Fig. 4).

## Discussion

In this work, we assessed the association between intratumoral *F. nucleatum* and clinico-pathological features and OS, RFS and MFS in two independent cohorts of patients with OSCC, and explored the interplay between *F. nucleatum* and well-known immune-related genes. Overall, we showed that *F. nucleatum* identified a subgroup of OSCC, more frequent in older, non-drinking patients, and associated with less frequent lymph node invasion and distant relapse, and favorable OS (independent predictor), RFS and MFS outcomes in the merged cohort. Other independent prognostic indicators included UICC stage and *TP53* mutational status, as previously reported^6^. Interestingly, the association of low pT or pN stage with *F. nucleatum* positivity allowed the identification of a patient subgroup with remarkably good prognosis.

The prevalence of *F. nucleatum* positivity in our study was higher than previously described (82% vs. 16% in a recently published meta-analysis), which may be explained by the high sensitivity of our real-time quantitative PCR assay and the selection of OSCC tumors only (in which *F. nucleatum* are enriched)^28^.

The positive correlation between *F. nucleatum* and survival was unexpected as this bacteria is usually associated with poor prognosis in cancers, particularly in colorectal cancer^29–32^. Based on several studies suggesting that *F. nucleatum* modulates the local immunity of cancers^18–20^, we assessed the association between *F. nucleatum* loads and expression of various immune-related genes to explore the underlying mechanisms of the favorable prognosis of *F. nucleatum* positive tumors. We showed that tumors with high *F. nucleatum* loads displayed low RNA levels of M2 macrophages (CD163), CD4 lymphocytes, fibroblasts (PDGFRß), TLR4, OX40 ligand (TNFSF4), and TNFRSF9, and high levels of TNFSF9 and IL-1ß. These results are consistent with our previously published work in which we identified high OX40 ligand and high PDGFRß as factors associated with poor survival^21^. Immunohistochemistry analyses confirmed that gram-negative (LPS-positive) bacteria (including *F. nucleatum*) load inversely correlated with CD163-positive cells.

Data regarding the effects of *F. nucleatum* on inflammation are conflicting. In most studies, *F. nucleatum* infection was shown to expand myeloid-derived immune cells and Tregs and promote M2 polarization of macrophages, inducing pro-tumoral inflammation, which inhibit T-cell proliferation and induce T-cell apoptosis^33, 34^. It also directly inhibits cytotoxic T-cell activities via proteins such as Fap2^35, 36^. TLRs are a family of receptors involved in the detection of microbial agents to induce activation of inflammatory and antimicrobial innate immune responses^37^. TLR4 is a receptor expressed at the surface of macrophages and tumor cells and has been involved in these pro-inflammatory/immunosuppressive activities of *F. nucleatum*^38, 39^. As an apparent paradox, in our study we observed that high *F. nucleatum* loads were associated with low levels of TLR4 and M2 macrophages. Interestingly, in a mice model of intestinal inflammation, TLR2/TLR4 knock-out induced increased colonization of *F. nucleatum* and production of pro-inflammatory cytokines including IL-1ß (as observed in our study)^18^.

On another hand, another work suggested that *F. nucleatum* enhanced the TNFSF9/IL-1ß signaling inducing M1 macrophage polarization^19^. This is consistent with the positive correlation that we observed between *F. nucleatum* loads and expression levels of TNFSF9 and IL-1ß cytokines. The lack of effect on M1 polarization could be explained by the concomitant decrease in TNFSF9 receptor.

It is noteworthy that inflammation and M2 infiltrates were found to be associated with poor prognosis in HNSCC^40–42^.

Taken together, these data suggest that in OSCC *F. nucleatum* may be associated with “permissive” tumor microenvironment, insensitive to pro-inflammatory signals, with low TLR4 signaling and low recruitment of M2, resulting in favorable clinical outcomes. Of note, defects in TLR functions have been associated with ageing, which may partially account for the higher proportion of older patients in the *F. nucleatum* positive group^43, 44^.

This work provides a new insight into the prognostic role of intratumoral *F. nucleatum* in OSCC patients and opens new avenues regarding the biological interplay between this bacteria and OSCC tumor immune microenvironment. Yet our study has some limitations. First, the small sample size did not allow reaching statistical significance in each individual cohort and merging the two cohorts was necessary to obtain sufficient statistical power for OS. In addition, the immune-related gene analysis was based on selected genes, which are not fully specific of each immune cell subtypes. Overall, these results would require further validation in larger prospective cohorts from randomized clinical trials and using more comprehensive methods such as RNA sequencing. Moreover, saliva samples and normal oral tissue were not available for analysis in our study; it could be of interest to assess the correlation between intratumoral *F. nucleatum* expression and saliva/normal tissue levels in further studies.

In conclusion, we highlight a unique association between *F. nucleatum* and OSCC patient survival and tumor immune microenvironment. This can give a rationale for further exploration of the role of *F. nucleatum* in OSCC carcinogenesis and response to treatment, particularly immune therapy.

## Supplementary Information

Supplementary Information 1.

Supplementary Table S1.

Supplementary Table S2.

Supplementary Table S3.

Supplementary Table S4.

Supplementary Table S5.

Supplementary Table S6.

Supplementary Table S7.

Supplementary Figure S1.

Supplementary Figure S2.

Supplementary Figure S3.

Supplementary Figure S4.

## Abbreviations

95%CI: 95% Confidence interval
*F. nucleatum*: *Fusobacterium nucleatum*
HNSCC: Head and neck squamous cell carcinoma
HPV: Human papillomavirus
HR: Hazard ratios
HRM: High resolution melting
HS: Histologic score
LPS: Lipopolysaccharide
MFS: Metastasis-free survival
NGS: Next generation sequencing
OS: Overall survival
OSCC: Oral squamous cell carcinoma
PCR: Polymerase chain reaction
RFS: Relapse-free survival
Treg: T regulatory cell
TLR: Toll-like receptor
UICC: Union for international cancer control
