# Supplementary material for: Prognostic value of intratumoral Fusobacterium nucleatum and association with immune-related gene expression in oral squamous cell carcinoma patients
Source: Sci Rep. 2021 Apr 12;11:7870. doi: 10.1038/s41598-021-86816-9 (PMC8041800; doi:10.1038/s41598-021-86816-9)
Supplement: Supplementary file 1 — Supplementary Information 1. [file 41598_2021_86816_MOESM1_ESM.docx]

# **Prognostic value of intratumoral** ***Fusobacterium nucleatum* and association with immune-related gene expression in oral squamous cell carcinoma patients**

Cindy Neuzillet, Manon Marchais, Sophie Vacher, Marc Hilmi, Anne Schnitzler, Didier Meseure, Renaud Leclere, Charlotte Lecerf, Coraline Dubot, Emmanuelle Jeannot, Jerzy Klijanienko, Odette Mariani, Valentin Calugaru, Caroline Hoffmann, Maria Lesnik, Nathalie Badois, Edith Borcoman, Eliane Piaggio, Maud Kamal, Christophe Le Tourneau, Ivan Bieche

**SUPPLEMENTARY MATERIAL**

**Supplementary Table 1. Nucleotide sequences of the primers used for PCR.**

**Supplementary Table 2. Relationship between *F. nucleatum* status and clinical, biological and pathological characteristics of the 122 patients of cohort #1.**

**Supplementary Table 3. Clinical, biological and pathological characteristics of the 122 patients of cohort #1, in relation with overall survival (OS).**

**Supplementary Table 4. Relationship between *F. nucleatum* status and clinical, biological and pathological characteristics of the 61 oral cavity patients (OSCC) of cohort #1.**

**Supplementary Table 5. Relationship between *F. nucleatum* status and clinical, biological and pathological characteristics of the 90 patients of cohort #2.**

**Supplementary Table 6. Relationship between *F. nucleatum* load and immune-related gene expression in 115 *F. nucleatum*-positive oral cavity tumors from the merged cohort.**

**Supplementary Table 7. Examples of representative cases showing the relationship between F. nucleatum DNA load and lipopolysaccharide (LPS) protein by immunohistochemistry and CD163 mRNA load and immunostaining assessed by Histologic Score (HS).**

**Supplementary Figure 1. Distribution of F. nucleatum loads (Nfn value) (A) in the cohort#1 (N=122) and (B) in the merged cohort (N=151).**

**Supplementary Figure 2. Overall survival according to *F. nucleatum* status (A) in the oropharynx tumor subgroup (n=22), (B) in the hypopharynx tumor subgroup (n=17), and (C) in the laxynx tumor subgroup (n=17) of the development set.**

**Supplementary Figure 3. Correlations between *F. nucleatum* load and *TNFSF4*, *TNFSF9*, *IL1B*, and *CD163* RNA expression levels.**

**Supplementary Figure 4. Representative pictures of lipopolysaccharide (LPS) immunostaining showing localization in the cytoplasm of tumor and immune cells (macrophagic and non-macrophagic) and in the form of extracellular bacterial vesicles.**
