# Supplementary material for: Prognostic value of intratumoral Fusobacterium nucleatum and association with immune-related gene expression in oral squamous cell carcinoma patients
Source: Sci Rep. 2021 Apr 12;11:7870. doi: 10.1038/s41598-021-86816-9 (PMC8041800; doi:10.1038/s41598-021-86816-9)
Supplement: Supplementary file 2 — Supplementary Table S1. [file 41598_2021_86816_MOESM2_ESM.docx]

**Supplementary Table 1. Nucleotide sequences of the primers used for PCR.**

| **Name** | **Gene ID** | **Forward primer 5’ to 3’** | **Reverse primer 5’ to 3’** | **Ct (average value in tumor samples)** | **Tm (°C)** |
| --- | --- | --- | --- | --- | --- |
| **TIGIT** | 201633 | CTCCCCTCGCCTCAGGAATGAT | CCGTGGTGGAGGAGAGGTGACA | 26.96 | 65 |
| **CTLA4** | 1493 | TCAGCTGAACCTGGCTACCAGGAC | GGGCCACGTGCATTGCTTTG | 28.95 | 65 |
| **PDCD1** | 5133 | TCGTCTGGGCGGTGCTACAAC | AGGGCCTGTCTGGGGAGTCTAAG | 29.67 | 65 |
| **CD274** | 29126 | GCTGAATTGGTCATCCCAGAACTAC | AAACGGAAGATGAATGTCAGTGCTAC | 26.46 | 65 |
| **PDCD1LG2** | 80380 | TCCTGCTAATGTTGAGCCTGGAA | GTCACATTGCTGCCATGCTCTATTAT | 26.32 | 65 |
| **TNFSF9** | 8744 | TGGTGGCCCAAAATGTTCTGCT | CTGCCAGGCCTGGGTCACTGTA | 30.17 | 65 |
| **TNFRSF9** | 3604 | CATATGCAGGCAGTGTAAAGGTGTT | ACCCTGGAGTGCAGTCACACTCT | 29.68 | 65 |
| **TNFSF18** | 8995 | CTTTGCTCCTTCAGTTGGCTAATCTT | GCCATTTGCCATTTTGAGGGTA | 31.56 | 65 |
| **TNFRSF18** | 8784 | AGACCCTTGCTGCACGACCTG | GCCAAAACTGAATTTCCCCTGG | 29.32 | 65 |
| **ICOS** | 29851 | ATGTGCAGCCTTTGTTGTAGTCTG | GTGCACACTGGATGAATACTTCTTTT | 28.65 | 65 |
| **ICOSLG** | 23308 | CTTCTGCAGCAGAACCTGACTGT | CGGTACTGACTGGATTCTCTGTGAT | 26.69 | 65 |
| **TNFRSF4** | 7293 | AAGCCCTGGACCAACTGCAC | TTGCGTCCGAGCTATTGCTG | 29.36 | 65 |
| **TNFSF4** | 7292 | ATCCTTTCCAACAAGTCATCCAGCA | CGAGGATACCGATGTGATACCATGAG | 30.36 | 65 |
| **LAG3** | 3902 | CCTTTCTCTGCTCCTTTTGGTGACT | AATCGTCTTGGTCGCCACTGTCT | 27.63 | 65 |
| **TIM3** | 84868 | CTGCTGCTACTACTTACAAGGTCCTC | GCAGGGCAGATAGGCATTCTG | 27.80 | 65 |
| **CD27** | 939 | GTGCACCGAGTGTGATCCTCTT | GGCCTCCAGCATCTCACTGAC | 27.20 | 65 |
| **CD28** | 940 | GTGGCCTTTATTATTTTCTGGGTGAG | GGCGGGGAGTCATGTTCATGT | 27.72 | 65 |
| **CD276** | 80381 | AGGAGAATGCAGGAGCTGAGGA | TCAGAGGCTGCAGGGCTGTC | 25.25 | 65 |
| **IDO1** | 3620 | TGTTTCACCAAATCCACGATCAT | CCTTCATACACCAGACCGTCTGAT | 25.50 | 65 |
| **TLR2** | 7097 | GGGTCATCATCAGCCTCTCCA | GCTGCCCTTGCAGATACCATT | 27.41 | 65 |
| **TLR4** | 7099 | GTTCTACATCAAATGCCCCTACTCA | TGCACCTGGTTGGATAAAGTTCA | 29.06 | 65 |
| **IL1B** | 3553 | GCAAAAAAGCTTGGTGATGTCTG | AAGGACATGGAGAACACCACTTGT | 26.23 | 65 |
| **TGFB1** | 7040 | GTCACCCGCGTGCTAATGGT | TTCTCGGAGCTCTGATGTGTTGA | 24.59 | 65 |
| **IL6** | 3569 | CAATCTGGATTCAATGAGGAGAC | CTCTGGCTTGTTCCTCACTACTC | 31.76 | 65 |
| **IL10** | 3586 | GGCGCTGTCATCGATTTCTTC | AGATGCCTTTCTCTTGGAGCTTATT | 30.46 | 65 |
| **CXCL8** | 3576 | CACCGGAAGGAACCATCTCACTGT | TCCTTGGCAAAACTGCACCTTCA | 24.28 | 65 |
| **STAT1** | 6772 | AGCATGAAATCAAGAGCCTGGA | ACCATTGGTCTCGTGTTCTCTGTT | 21.86 | 65 |
| **IFNG** | 3458 | GAGTGTGGAGACCATCAAGGAAGA | GCGACAGTTCAGCCATCACTTG | 32.43 | 65 |
| **TNFA** | 7124 | GCCCAGGCAGTCAGATCATCTT | CCTCAGCTTGAGGGTTTGCTACA | 29.63 | 65 |
| **CXCL10** | 3627 | CTGACTCTAAGTGGCATTCAAGGAG | GGTTGATTACTAATGCTGATGCAGG | 23.31 | 65 |
| **CCL5** | 6352 | GCCCACATCAAGGAGTATTTCTACA | TTCGGGTGACAAAGACGACTG | 24.83 | 65 |
| **CXCR6** | 10663 | GGTTCAGCAGTTTCAATGACAGCA | CAGACCACAGACAAACACCACCAG | 26.80 | 65 |
| **CCR7** | 1236 | GGGGAAACCAATGAAAAGCGT | ATCTTGACACAGGCATACCTGGAA | 29.89 | 65 |
| **CXCL9** | 4283 | ATCCACCTACAATCCTTGAAAGAC | TCCATTCTTCAGTGTAGCAATGATTT | 25.03 | 65 |
| **IL3RA** | 3563 | ATCGCAAATTTCGCTATGAGCTT | GGAGGTTCTGTCTCTGACCTGTTCT | 27.59 | 65 |
| **NKG7** | 4818 | CCCCAGATCCAGACCTTCTTCTC | CCAGGCTCAGGGCACCTGTA | 27.09 | 65 |
| **CMKLR1** | 1240 | TCAACCTGGCAGTGGCAGAT | CCCGAAAACCCAGTGGTAGTC | 26.60 | 65 |
| **CD3E** | 916 | AAGATGGTAATGAAGAAATGGGTGGT | TGAGGGCATGTCAATATTACTGTGGT | 26.59 | 65 |
| **CD8A** | 925 | CCGGTCTTCCTGCCAGCGAAG | GGCGCCGGTGTTGGTGGTC | 27.47 | 65 |
| **FOXP3** | 50943 | TGGCCCGGATGTGAGAAGGTC | CGCCTGGCAGTGCTTGAGGA | 29.08 | 65 |
| **MS4A1** | 931 | CAACTGTGAACCAGCTAATCCCTCTG | GCATCACTGACAAAATGCCCAAGA | 28.20 | 65 |
| **PDGFRB** | 5155 | CCCCAGTGCCGAGTTAGAAGAC | GCACGTAGCCGCTCTCAACC | 24.29 | 65 |
| **FUT4** | 2526 | CTGCCATGGACCGTCTGTGT | CCCCAGCAAGCGTAGGTGA | 30.23 | 65 |
| **CD14** | 929 | CCGGGTGCCGCTGTGTAGAAA | AGGACGCGCGCTCCATGGT | 25.76 | 65 |
| **GZMA** | 3001 | GGAAGAGACTCGTGCAATGGAGAT | GCATTTATTTTCAAGGCCAAAGGA | 28.80 | 65 |
| **GZMB** | 3002 | CGCCATTATTACGACAGTACCATTGA | GGCCTCCAGAGTCCCCCTTAA | 27.85 | 65 |
| **CD4** | 920 | AGGCGGTGTGGGTGCTGAA | GACCATGTGGGCAGAACCTTGA | 27.42 | 65 |
| **ITGAX** | 3687 | CCATGCACAGATACCAGGTCAAT | CTCCACAGGCACCCAGAAGTT | 27.86 | 65 |
| **CD1C** | 911 | GACAATGCAGACGCATCCCA | CAACTCGTCCAGCCATCCTGA | 29.30 | 65 |
| **CD80** | 941 | ACACACGGAGGCAGGGAACAT | GGTCACGTGGATAACACCTGAACA | 28.37 | 65 |
| **CD86** | 942 | TCTGAACTGTCAGTGCTTGCTAACTT | AGGTTCTGGGTAACCGTGTATAGATG | 27.90 | 65 |
| **CD163** | 9332 | TGCTGTGGCCTGCATAGAGAGT | CCCAGGAGCCCTCATGATAGAT | 27.41 | 65 |
| **ITGAM** | 3684 | GCATCCGCAAAGTGGTACGA | TTTTCTCCATCCGTGATGACAACT | 30.37 | 65 |
| **APOBEC3A** | 200315 | GACAATGGCACCTCGGTCAAGA | GGGTCCAACTGCAAAGAAGGAAC | 26.94 | 65 |
| **APOBEC3B** | 9582 | ATTCCTGCACCGCACGCTA | GTCGAAGGACCAAAGGGTCATTA | 27.96 | 65 |
| **MKI67** | 4288 | ATTGAACCTGCGGAAGAGCTGA | GGAGCGCAGGGATATTCCCTTA | 24.18 | 65 |
| **CCND1** | 595 | GGATGCTGGAGGTCTGCGA | AGAGGCCACGAACATGCAAG | 23.52 | 65 |
| **VIM** | 7431 | TCAGACAGGATGTTGACAATGCGT | CTGCAGCTCCTGGATTTCCTCTT | 20.39 | 65 |
| **CDH1** | 999 | CGCATTGCCACATACACTCTCTT | TCGGGCTTGTTGTCATTCTGAT | 23.37 | 65 |
| **MMP9** | 4318 | CGGCTTGCCCTGGTGCAGT | CGTCCCGGGTGTAGAGTCTCTCG | 24.38 | 65 |
| **MMP1** | 4312 | GGCTTGAAGCTGCTTACGAATTT | ACAGCCCAGTACTTATTCCCTTTGA | 22.32 | 65 |
| **DNTP63** | 8626 | GGAAAACAATGCCCAGACTCAAT | TGTTCAGGAGCCCCAGGTTC | 22.42 | 65 |
| **TATP63** | 8626 | AGATTAGCATGGACTGTATCCGCA | GAGCCCCAGGTTCGTGTACTGT | 29.10 | 65 |
| **TPX2** | 22974 | GACTTGGAAGCACCAGCTGGA | GGTGTTTGGACGAGCCTTGAA | 25.09 | 65 |
