# Supplementary material for: Prognostic value of intratumoral Fusobacterium nucleatum and association with immune-related gene expression in oral squamous cell carcinoma patients
Source: Sci Rep. 2021 Apr 12;11:7870. doi: 10.1038/s41598-021-86816-9 (PMC8041800; doi:10.1038/s41598-021-86816-9)
Supplement: Supplementary file 3 — Supplementary Table S2. [file 41598_2021_86816_MOESM3_ESM.doc]

**Supplementary Table 2. Relationship between *F. nucleatum* status and clinical, biological and pathological characteristics of the 122 patients of cohort #1.**

|  | **Patients (%)** | **Number of patients (%)** | | ***p*-value**^a^ |
| --- | --- | --- | --- | --- |
|  |  | ***F. nucleatum* negative** | ***F. nucleatum* positive** |  |
| *Total* | 122 (100) | 35 (28.7) | 87 (71.3) |  |
| *Age*  <56  ≥56 | 58 (47.5)  64 (52.5) | 15 (42.9)  20 (57.1) | 43 (49.4)  44 (50.6) | 0.51 (NS) |
| *Sex*  Female  Male | 29 (23.8)  93 (76.2) | 4 (11.4)  31 (88.6) | 25 (28.7)  62 (71.3) | **0.042 *** |
| *Alcohol ^b^*  No  Yes | 31 (35.2)  57 (64.8) | 3 (13.6)  19 (86.4) | 28 (42.4)  38 (57.6) | **0.014 *** |
| *Tobacco ^c^*  No  Yes | 29 (28.7)  72 (71.3) | 5 (17.2)  24 (82.8) | 24 (33.3)  48 (66.7) | 0.11 (NS) |
| *Alcohol and tobacco ^b^*  No  Yes | 36 (40.9)  52 (59.1) | 4 (18.2)  18 (81.8) | 32 (48.5)  34 (51.5) | **0.012 *** |
| *pT*  1  2  3  4 | 17 (13.9)  32 (26.2)  26 (21.3)  47 (38.5) | 3 (8.6)  8 (22.9)  9 (25.7)  15 (42.9) | 14 (16.1)  24 (27.6)  17 (19.5)  32 (36.8) | 0.59 (NS) |
| *pN*  0  1  2  3 | 75 (61.5)  14 (11.5)  22 (18.0)  11 (9.0) | 14 (40.0)  3 (8.6)  11 (31.4)  7 (20.0) | 61 (70.1)  11 (12.6)  11 (12.6)  4 (4.6) | **0.0014 **** |
| *HPV*  Negative  Positive | 113 (92.6)  9 (7.4) | 31 (88.6)  4 (11.4) | 82 (94.3)  5 (5.7) | 0.28 (NS) |
| *UICC stage*  Stage I  Stage II  Stage III  Stage IV | 16 (13.1)  22 (18)  22 (18)  62 (50.8) | 2 (5.7)  3 (8.6)  6 (17.1)  24 (68.6) | 14 (16.1)  19 (21.8)  16 (18.4)  38 (43.7) | 0.06 (NS) |
| *Tumor location*  Oral cavity  Oropharynx  Hypopharynx  Larynx | 61 (50)  22 (18)  17 (14)  22 (18) | 13 (37.1)  3 (8.6)  12 (34.3)  7 (20) | 48 (55.2)  19 (21.8)  5 (5.7)  15 (17.2) | **0.0003 ***** |
| *TP53 mutational status*  Wild-type  Mutated | 50 (41)  72 (59) | 13 (37.1)  22 (62.9) | 37 (42.5)  50 (57.5) | 0.58 (NS) |
| *PIK3CA mutational status* |  |  |  |  |
| Wild-type | 107 (87.7) | 31 (88.6) | 76 (87.4) | 0.78 (NS) |
| Mutated | 15 (12.3) | 4 (11.4) | 11 (12.6) |  |
| Relapse  No  Yes | 85 (69.7)  37 (30.3) | 23 (65.7)  12 (34.3) | 62 (71.3)  25 (28.7) | 0.55 (NS) |
| Locoregional relapse  Distant metastasis  Both | 15 (40.5)  22 (59.5)  0 (0) | 5 (41.7)  7 (58.3)  0 (0) | 10 (40.0)  15 (60.0)  0 (0) | 0.79 (NS) |

^a^ Chi-square test, Chi-square test with Yates’ correction or Fisher test if appropriate

^b^ Information available for 88 patients

^c^ Information available for 101 patients

*: P <0.05

**: P <0.01

***: P <0.001

HPV: human papilloma virus; UICC: Union for International Cancer Control; NS: Not Significant
