# Supplementary material for: Prognostic value of intratumoral Fusobacterium nucleatum and association with immune-related gene expression in oral squamous cell carcinoma patients
Source: Sci Rep. 2021 Apr 12;11:7870. doi: 10.1038/s41598-021-86816-9 (PMC8041800; doi:10.1038/s41598-021-86816-9)
Supplement: Supplementary file 4 — Supplementary Table S3. [file 41598_2021_86816_MOESM4_ESM.doc]

**Supplementary Table 3. Clinical, biological and pathological characteristics of the 122 patients of cohort #1, in relation with overall survival (OS).**

|  | **Patients (%)** | **Death (%)** | **HR^a^** | **95% CI^b^** | **OS**^c^ |
| --- | --- | --- | --- | --- | --- |
| *Total* | 122 (100) | 62 (50.8) |  |  |  |
| *Age*  <56  ≥56 | 58 (47.5)  64 (52.5) | 30 (51.7)  32 (50.0) | 1  0.75 | 0.45-1.25 | 0.26 (NS) |
| *Sex*  Female  Male | 29 (23.8)  93 (76.2) | 13 (44.8)  49 (52.7) | 1  1.22 | 0.68-2.18 | 0.52 (NS) |
| *Alcohol^d^*  No  Yes | 31 (35.2)  57 (64.8) | 10 (32.3)  32 (56.1) | 1  2.26 | 1.23-4.18 | **0.019 *** |
| *Tobacco^e^*  No  Yes | 29 (28.7)  72 (71.3) | 8 (27.6)  40 (55.6) | 1  2.42 | 1.32-4.43 | **0.017 *** |
| *Alcohol and tobacco ^b^*  No  Yes | 36 (40.9)  52 (59.1) | 13 (31.0)  29 (69.0) | 1  1.85 | 1.00-3.40 | 0.060 (NS) |
| *pT*  1  2  3  4 | 17 (13.9)  32 (26.2)  26 (21.3)  47 (38.5) | 5 (8.1)  19 (30.6)  9 (14.5)  29 (46.8) | 1  2.96  1.75  3.39 | 1.32-6.63  0.61-4.99  1.69-6.81 | **0.021 ***  **0.022 ***  0.30 (NS)  **0.0064 **** |
| *pN*  0  1  2  3 | 75 (61.5)  14 (11.5)  22 (18.0)  11 (9.0) | 34 (54.8)  9 (14.5)  11 (17.7)  8 (12.9) | 1  1.90  1.57  2.69 | 0.76-4.73  0.72-3.4  0.88-8.18 | **0.045 ***  0.080 (NS)  0.19 (NS)  **0.0085 **** |
| *HPV*  Negative  Positive | 113 (92.6)  9 (7.4) | 62 (54.9)  0 (0) | Undefined |  | **0.0061 **** |
| *UICC stage*  Stage I  Stage II  Stage III  Stage IV | 16 (13.1)  22 (18)  22 (18)  62 (50.8) | 5 (31.3)  14 (63.6)  7 (31.8)  36 (58.1) | 1  3.40  1.40  3.14 | 1.38-8.39  0.45-4.34  1.61-6.10 | **0.013 ***  **0.011***  0.56 (NS)  **0.010 **** |
| *Tumor location*  Oral cavity  Oropharynx  Hypopharynx  Larynx | 61 (50)  22 (18)  17 (14)  22 (18) | 31 (50.8)  7 (31.8)  12 (70.6)  12 (54.5) | 1  0.73  1.82  1.69 | 0.35-1.56  0.83-4.00  0.79-3.65 | 0.098 (NS)  0.45 (NS)  0.071 (NS)  0.11 (NS) |
| *TP53 mutational status*  Wild-type  Mutated | 50 (41)  72 (59) | 16 (32)  46 (63.9) | 1  2.56 | 1.55-4.21 | **0.0007***** |
| *PIK3CA mutational status*  Wild-type  Mutated | 107 (87.7)  15 (12.3) | 54 (50.5)  8 (53.3) | 1  1.12 | 0.52-2.44 | 0.76 (NS) |
| Relapse  No  Yes | 85 (62.3)  37 (37.7) | 38 (44.7)  24 (64.9) | 1  1.31 | 0.77-2.23 | 0.30 (NS) |
| Locoregional relapse  Distant metastasis  Both | 15 (40.5)  22 (59.5)  0 (0) | 7 (46.7)  17 (77.3) | 1  2.95 | 1.32-6.58 | **0.0085 **** |

^a^ Hazard ratio (logrank)

^b^ 95% Confidence Interval

^c^ Log-rank test

^d^ Information available for 88 patients

^e^ Information available for 101 patients

*: P <0.05

**: P <0.01

***: P <0.001

OS: overall survival; NS: not significant; HPV: human papilloma virus; UICC: Union for International Cancer Control; HR: Hazard Ratio
