# Supplementary material for: Prognostic value of intratumoral Fusobacterium nucleatum and association with immune-related gene expression in oral squamous cell carcinoma patients
Source: Sci Rep. 2021 Apr 12;11:7870. doi: 10.1038/s41598-021-86816-9 (PMC8041800; doi:10.1038/s41598-021-86816-9)
Supplement: Supplementary file 5 — Supplementary Table S4. [file 41598_2021_86816_MOESM5_ESM.doc]

**Supplementary Table 4. Relationship between *F. nucleatum* status and clinical, biological and pathological characteristics of the 61 oral cavity patients (OSCC) of cohort #1.**

|  | **Patients (%)** | **Number of patients (%)** | | ***p*-value**^a^ |
| --- | --- | --- | --- | --- |
|  |  | ***F. nucleatum* negative** | ***F. nucleatum* positive** |  |
| *Total* | 61 (100) | 13 (21.3) | 48 (78.7) |  |
| *Age*  <56  ≥56 | 24 (39.3)  37 (60.7) | 5 (38.5)  8 (61.5) | 19 (39.6)  29 (60.4) | 0.94 (NS) |
| *Sex*  Female  Male | 24 (39.3)  37 (60.7) | 2 (15.4)  11 (84.6) | 22 (45.8)  26 (54.2) | 0.13 (NS) |
| *Alcohol ^b^*  No  Yes | 22 (45.8)  26 (54.2) | 1 (12.5)  7 (87.5) | 21 (52.5)  19 (47.5) | 0.092 (NS) |
| *Tobacco ^c^*  No  Yes | 20 (38.5)  32 (61.5) | 2 (18.2)  9 (81.8) | 18 (43.9)  23 (56.1) | 0.23 (NS) |
| *Alcohol and tobacco ^b^*  No  Yes | 25 (52.1)  23 (47.9) | 2 (25.0)  6 (75.0) | 23 (57.5)  17 (42.5) | 0.20 (NS) |
| *pT*  1  2  3  4 | 14 (23.0)  14 (23.0)  21 (34.4)  12 (19.7) | 3 (23.1)  3 (23.1)  7 (53.8)  0 (0) | 11 (22.9)  11 (22.9)  14 (29.2)  12 (25.0) | 0.17 (NS) |
| *pN*  0  1  2  3 | 34 (55.7)  5 (8.2)  10 (16.4)  12 (19.7) | 5 (38.5)  0 (0)  2 (15.4)  6 (46.2) | 29 (60.4)  5 (10.4)  8 (16.7)  6 (12.5) | **0.043 *** |
| *Differentiation ^d^*  Verrucous  Grade I  Grade II  Grade III  Grade IV | 0 (0)  43 (76.8)  8 (14.3)  5 (8.9)  0 (0) | 0 (0)  8 (66.7)  2 (16.7)  2 (16.7)  0 (0) | 0 (0)  35 (79.5)  6 (13.6)  3 (6.8)  0 (0) | 0.53 (NS) |
| *Margins* ^d^  Negative or close  Positive | 44 (78.6)  12 (21.4) | 8 (66.7)  4 (33.3) | 36 (81.8)  8 (18.2) | 0.26 (NS) |
| *HPV*  Negative  Positive | 60 (98.4)  1 (1.6) | 12 (92.3)  1 (7.7) | 48 (100)  0 (0) | 0.21 (NS) |
| *UICC stage*  Stage I  Stage II  Stage III  Stage IV | 15 (24.6)  19 (31.1)  9 (14.8)  18 (29.5) | 2 (15.4)  2 (15.4)  4 (30.8)  5 (38.5) | 13 (27.1)  17 (35.4)  5 (10.4)  13 (27.1) | 0.12 (NS) |
| *TP53 mutational status*  Wild-type  Mutated | 27 (44.3)  34 (55.7) | 4 (30.8)  9 (69.2) | 23 (47.9)  25 (52.1) | 0.27 (NS) |
| *PIK3CA mutational status* |  |  |  |  |
| Wild-type | 54 (88.5) | 11 (84.6) | 43 (89.6) | 0.63 (NS) |
| Mutated | 7 (11.5) | 2 (15.4) | 5 (10.4) |  |
| Relapse  No  Yes | 36 (59.0)  25 (41.0) | 7 (53.9)  6 (46.2) | 29 (60.4)  19 (39.6) | 0.67 (NS) |
| Locoregional relapse  Distant metastasis  Both | 17 (68.0)  6 (24.0)  2 (8.0) | 3 (50.0)  3 (50.0)  0 (0) | 14 (73.7)  3 (15.8)  2 (10.5) | 0.20 (NS) |

^a^ Chi-square test, Chi-square test with Yates’ correction or Fisher test if appropriate

^b^ Information available for 48 patients

^c^ Information available for 52 patients

^d^ Information available for 56 patients

*: P <0.05

HPV: human papilloma virus; UICC: Union for International Cancer Control; NS: Not significant.
