# Supplementary material for: Prognostic value of intratumoral Fusobacterium nucleatum and association with immune-related gene expression in oral squamous cell carcinoma patients
Source: Sci Rep. 2021 Apr 12;11:7870. doi: 10.1038/s41598-021-86816-9 (PMC8041800; doi:10.1038/s41598-021-86816-9)
Supplement: Supplementary file 6 — Supplementary Table S5. [file 41598_2021_86816_MOESM6_ESM.doc]

**Supplementary Table 5. Relationship between *F. nucleatum* status and clinical, biological and pathological characteristics of the 90 patients of cohort #2.**

|  | **Patients (%)** | **Number of patients (%)** | | ***p*-value**^a^ |
| --- | --- | --- | --- | --- |
|  |  | ***F. nucleatum* negative** | ***F. nucleatum* positive** |  |
| *Total* | 90 (100) | 14 (15.6) | 76 (84.4) |  |
| *Age*  <56  ≥56 | 21 (23.3)  69 (76.7) | 8 (57.1)  6 (42.9) | 13 (17.1)  63 (82.9) | **0.0036 **** |
| *Sex*  Female  Male | 34 (37.8)  56 (62.2) | 5 (35.7)  9 (64.3) | 29 (38.2)  47 (61.8) | 0.86 (NS) |
| *Alcohol ^b^*  No  Yes | 57 (70.4)  24 (29.6) | 7 (58.3)  5 (41.7) | 50 (72.5)  19 (27.5) | 0.52 (NS) |
| *Tobacco ^c^*  No  Yes | 42 (48.8)  44 (51.2) | 5 (35.7)  9 (64.3) | 37 (51.4)  35 (48.6) | 0.28 (NS) |
| *Alcohol and tobacco ^b^*  No  Yes | 59 (72.8)  22 (27.2) | 7 (58.3)  5 (41.7) | 52 (75.4)  17 (24.6) | 0.38 (NS) |
| *pT*  1  2  3  4 | 7 (7.8)  24 (26.7)  37 (41.1)  22 (24.4) | 0 (0)  4 (28.6)  7 (50.0)  3 (21.4) | 7 (9.2)  20 (26.3)  30 (39.5)  19 (25.0) | 0.64 (NS) |
| *pN*  0  1  2  3 | 47 (52.2)  10 (11.1)  13 (14.4)  20 (22.2) | 4 (28.6)  0 (0)  4 (28.6)  6 (42.9) | 43 (56.6)  10 (13.2)  9 (11.8)  14 (18.4) | **0.028 *** |
| *Differentiation ^d^*  Verrucous  Grade I  Grade II  Grade III  Grade IV | 4 (4.5)  63 (70.8)  19 (21.3)  3 (3.4)  0 (0) | 0 (0)  11 (78.6)  1 (7.1)  2 (14.3)  0 (0) | 4 (5.3)  52 (69.3)  18 (24.0)  1 (1.3)  0 (0) | **0.040 *** |
| *Margins*  Negative or close  Positive | 78 (86.7)  12 (13.3) | 12 (85.7)  2 (14.3) | 66 (86.8)  10 (13.2) | >0.99 (NS) |
| *HPV*  Negative  Positive | 87 (96.7)  3 (3.3) | 13 (92.9)  1 (7.1) | 74 (97.4)  2 (2.6) | 0.40 (NS) |
| *UICC stage*  Stage I  Stage II  Stage III  Stage IV | 5 (5.6)  18 (20)  21 (23.3)  46 (51.1) | 0 (0)  2 (14.3)  2 (14.3)  10 (71.4) | 5 (6.6)  16 (21.1)  19 (25)  36 (47.4) | 0.37 (NS) |
| *TP53 mutational status*  Wild-type  Mutated | 62 (68.9)  28 (31.1) | 10 (71.4)  4 (28.6) | 52 (68.4)  24 (31.6) | 0.93 (NS) |
| *PIK3CA mutational status*  Mutated  Wild-type | 79 (87.8)  11 (12.2) | 12 (85.7)  2 (14.3) | 67 (88.2)  9 (11.8) | 0.68 (NS) |
| Relapse  No  Yes | 57 (63.3)  33 (36.7) | 6 (42.9)  8 (57.1) | 51 (67.1)  25 (32.9) | 0.084 (NS) |
| Locoregional relapse  Distant metastasis  Both | 21 (63.6)  5 (15.2)  7 (21.2) | 4 (50.0)  3 (37.5)  1 (12.5) | 17 (68.0)  2 (8.0)  6 (24.0) | 0.12 (NS) |
|  |  |  |  |  |

^a^ Chi-square test, Chi-square test with Yates’ correction or Fisher test if appropriate

^b^ Information available for 81 patients

^c^ Information available for 86 patients

^d^ Information available for 89 patients

*: P <0.05

**: P <0.01

HPV: human papilloma virus; UICC: Union for International Cancer Control; NS: Not Significant.
