# Supplementary material for: Prognostic value of intratumoral Fusobacterium nucleatum and association with immune-related gene expression in oral squamous cell carcinoma patients
Source: Sci Rep. 2021 Apr 12;11:7870. doi: 10.1038/s41598-021-86816-9 (PMC8041800; doi:10.1038/s41598-021-86816-9)
Supplement: Supplementary file 7 — Supplementary Table S6. [file 41598_2021_86816_MOESM7_ESM.docx]

**Supplementary Table 6. Relationship between *F. nucleatum* load and immune-related gene expression in 115 *F. nucleatum*-positive oral cavity tumors from the merged cohort.**

| **Gene** | **Alias** | **Cellular specificity** | ***F. nucleatum*** | | | **HR^c^** | **95% CI^d^** | **OS^e^** |  |
| --- | --- | --- | --- | --- | --- | --- | --- | --- | --- |
|  |  |  | **r^a^** | **p-value^a^** | **p-value corrected^b^** |  |  |  |  |
| **Checkpoint T cell and tumor cell genes** | | | | | | | | | |
| *TIGIT* |  |  | -0.270 | **0.0035** | 0.23 (NS) | 0.84 | 0.50-1.41 | 0.51 (NS) |  |
| *CTLA4* | *CD152* |  | -0.265 | **0.0042** | 0.28 (NS) | 0.51 | 0.30-0.86 | **0.014** * |  |
| *PDCD1* | *PD-1, CD279* |  | -0.245 | **0.0084** | 0.56 (NS) | 0.68 | 0.40-1.14 | 0.15 (NS) |  |
| *CD274* | *PDL1* |  | -0.103 | 0.27 (NS) | 1.00 (NS) | 0.65 | 0.39-1.10 | 0.11 (NS) |  |
| *PDCD1LG2* | *PDL2* |  | -0.156 | 0.097 (NS) | 1.00 (NS) | 1.04 | 0.62-1.76 | 0.88 (NS) |  |
| *TNFSF9* | *CD137L* |  | +0.312 | **0.0007** | **0.047** | 1.03 | 0.61-1.74 | 0.92 (NS) |  |
| *TNFRSF9* | *CD137* |  | -0.360 | **<0.0001** | **0.0067** | 0.87 | 0.51-1.46 | 0.59 (NS) |  |
| *TNFSF18* | *GITRL* |  | -0.108 | 0.25 (NS) | 1.00 (NS) | 0.48 | 0.29-0.82 | **0.0068** ** |  |
| *TNFRSF18* | *GITR* |  | -0.006 | 0.95 (NS) | 1.00 (NS) | 0.88 | 0.52-1.49 | 0.64 (NS) |  |
| *ICOS* |  |  | -0.252 | **0.0065** | 0.44 (NS) | 0.73 | 0.43-1.24 | 0.24 (NS) |  |
| *ICOSLG* |  |  | -0.266 | **0.004** | 0.27 (NS) | 1.03 | 0.61-1.74 | 0.91 (NS) |  |
| *TNFRSF4* | *OX40* |  | -0.219 | **0.019** | 1.00 (NS) | 0.83 | 0.49-1.40 | 0.48 (NS) |  |
| *TNFSF4* | *OX40L* |  | -0.401 | **<0.0001** | **0.0067** | 1.11 | 0.65-1.87 | 0.70 (NS) |  |
| *LAG3* | *CD223* |  | -0.165 | 0.078 (NS) | 1.00 (NS) | 0.71 | 0.42-1.20 | 0.20 (NS) |  |
| *TIM3* | *HAVCR2* |  | -0.295 | **0.0014** | 0.094 (NS) | 0.98 | 0.58-1.65 | 0.93 (NS) |  |
| *CD27* | *TNFRSF7* |  | -0.297 | **0.0012** | 0.080 (NS) | 0.65 | 0.38-1.09 | 0.11 (NS) |  |
| *CD28* |  |  | -0.306 | **0.0009** | 0.060 (NS) | 1.06 | 0.63-1.80 | 0.83 (NS) |  |
| *CD276* | *B7H3* |  | -0.043 | 0.65 (NS) | 1.00 (NS) | 2.20 | 1.29-3.74 | **0.0029** ** |  |
| *IDO1* | *IDO* |  | -0.090 | 0.34 (NS) | 1.00 (NS) | 0.48 | 0.28-0.82 | **0.0069** ** |  |
| **Chemokine genes** | | | | | | | | | |
| *TLR2* |  |  | -0.120 | 0.20 (NS) | 1.00 (NS) | 1.23 | 0.73-2.09 | 0.43 (NS) |  |
| *TLR4* |  |  | -0.329 | **0.0003** | **0.020** | 0.91 | 0.54-1.53 | 0.72 (NS) |  |
| *IL1B* |  |  | +0.385 | **<0.0001** | **0.0067** | 0.76 | 0.45-1.29 | 0.31 (NS) |  |
| *TGFB1* |  |  | -0.047 | 0.62 (NS) | 1.00 (NS) | 1.57 | 0.93-2.66 | 0.088 (NS) |  |
| *IL6* |  |  | +0.178 | 0.058 (NS) | 1.00 (NS) | 1.24 | 0.74-2.10 | 0.41 (NS) |  |
| *IL10* |  |  | -0.079 | 0.40 (NS) | 1.00 (NS) | 0.88 | 0.52-1.49 | 0.64 (NS) |  |
| *CXCL8* | *IL8* |  | +0.302 | **0.0011** | 0.074 (NS) | 1.31 | 0.77-2.21 | 0.31 (NS) |  |
| *STAT1* |  |  | -0.077 | 0.4128 (NS) | 1.00 (NS) | 0.52 | 0.31-0.89 | **0.014** * |  |
| *IFNG* |  |  | -0.118 | 0.21 (NS) | 1.00 (NS) | 0.45 | 0.27-0.76 | **0.0031** ** |  |
| *TNFA* | *TNF* |  | +0.009 | 0.92 (NS) | 1.00 (NS) | 0.95 | 0.56-1.60 | 0.84 (NS) |  |
| *CXCL10* | *IP10* |  | -0.028 | 0.77 (NS) | 1.00 (NS) | 0.48 | 0.29-0.82 | **0.0069** ** |  |
| *CCL5* | *RANTES* |  | -0.053 | 0.57 (NS) | 1.00 (NS) | 0.80 | 0.47-1.34 | 0.39 (NS) |  |
| *CXCR6* |  |  | -0.266 | **0.0041** | 0.27 (NS) | 0.79 | 0.47-1.33 | 0.39 (NS) |  |
| *CCR7* |  |  | -0.185 | **0.048** | 1.00 (NS) | 0.65 | 0.39-1.10 | 0.11 (NS) |  |
| *CXCL9* | *MIG* |  | -0.155 | 0.098 (NS) | 1.00 (NS) | 0.74 | 0.44-1.24 | 0.25 (NS) |  |
| *IL3RA^f^* | *CD123* |  | -0.203 | **0.030** | 1.00 (NS) | 0.63 | 0.37-1.06 | 0.085 (NS) |  |
| **Immune cell population genes** | | | | | | | | | |
| *NKG7* |  | NK | -0.226 | **0.015** | 1.00 (NS) | 0.70 | 0.41-1.18 | 0.18 (NS) |  |
| *CMKLR1* | *ChemR23* | NK | -0.299 | **0.0012** | 0.080 (NS) | 1.01 | 0.60-1.71 | 0.97 (NS) |  |
| *CD3E* | *CD3* | LT | -0.271 | **0.0034** | 0.23 (NS) | 0.72 | 0.42-1.21 | 0.21 (NS) |  |
| *CD8A* | *CD8* | LTc | -0.294 | **0.0014** | 0.094 (NS) | 0.94 | 0.55-1.58 | 0.81 (NS) |  |
| *FOXP3* |  | Treg | -0.302 | **0.0011** | 0.074 (NS) | 1.12 | 0.66-1.89 | 0.68 (NS) |  |
| *MS4A1* | *CD20* | LB | -0.323 | **0.0004** | **0.027** | 1.02 | 0.61-1.73 | 0.93 (NS) |  |
| *PDGFRB* |  | Fibroblast | -0.347 | **0.0001** | **0.0067** | 2.08 | 1.23-3.53 | **0.0062** ** |  |
| *FUT4* | *CD15* | Neutrophil | -0.249 | **0.0072** | 0.48 (NS) | 1.33 | 0.78-2.25 | 0.29 (NS) |  |
| *CD14* |  | Macrophage | -0.216 | **0.021** | 1.00 (NS) | 0.73 | 0.43-1.23 | 0.24 (NS) |  |
| *GZMA* |  | LT/NK | -0.219 | **0.019** | 1.00 (NS) | 0.70 | 0.42-1.18 | 0.19 (NS) |  |
| *GZMB* |  | LT/NK | -0.134 | 0.15 (NS) | 1.00 (NS) | 0.63 | 0.37-1.06 | 0.085 (NS) |  |
| *CD4* |  | LT helper | -0.342 | **0.0002** | **0.013** | 1.02 | 0.60-1.72 | 0.95 (NS) |  |
| *ITGAX* | *CD11C* | Dentritic cell | -0.265 | **0.0041** | 0.27 (NS) | 0.78 | 0.46-1.31 | 0.34 (NS) |  |
| *CD1C^c^* | *BDCA1* | Dentritic cell | -0.309 | **0.0008** | 0.054 (NS) | 0.99 | 0.58-1.67 | 0.96 (NS) |  |
| *CD80* | *B7-1* | M1 | -0.245 | **0.0084** | 0.56 (NS) | 0.87 | 0.51-1.46 | 0.59 (NS) |  |
| *CD86* | *B7-2* | M1/LB | -0.293 | **0.0015** | 0.10 (NS) | 0.75 | 0.44-1.26 | 0.27 (NS) |  |
| *CD163* |  | M2 | -0.331 | **0.0003** | **0.020** | 1.04 | 0.62-1.76 | 0.88 (NS) |  |
| *ITGAM* | *CD11B* | MDSC | -0.305 | **0.0009** | 0.060 (NS) | 0.89 | 0.53-1.50 | 0.66 (NS) |  |
| **APOBEC genes** | | | | | | | | | |
| *APOBEC3A* |  |  | +0.108 | 0.25 (NS) | 1.00 (NS) | 0.98 | 0.58-1.65 | 0.93 (NS) |  |
| *APOBEC3B* |  |  | +0.028 | 0.77 (NS) | 1.00 (NS) | 0.99 | 0.59-1.68 | 0.98 (NS) |  |
| **Cell proliferation genes** | | | | | | | | | |
| *MKI67* | *KI-67* |  | +0.034 | 0.72 (NS) | 1.00 (NS) | 0.84 | 0.50-1.42 | 0.52 (NS) |  |
| *CCND1* |  |  | +0.065 | 0.49 (NS) | 1.00 (NS) | 1.43 | 0.84-2.41 | 0.19 (NS) |  |
| **EMT genes** | | | | | | | | | |
| *VIM* |  |  | -0.281 | **0.0024** | 0.16 (NS) | 1.35 | 0.80-2.29 | 0.26 (NS) |  |
| *CDH1* | *E-CADH* |  | +0.187 | **0.046** | 1.00 (NS) | 0.67 | 0.40-1.14 | 0.14 (NS) |  |
| **Differentiation/invasion genes** | | | | | | | | | |
| *MMP9* |  |  | -0.100 | 0.29 (NS) | 1.00 (NS) | 1.59 | 0.94-2.69 | 0.081 (NS) |  |
| *MMP1* |  |  | +0.105 | 0.27 (NS) | 1.00 (NS) | 1.21 | 0.72-2.04 | 0.47 (NS) |  |
| *DNTP63* |  |  | +0.241 | **0.0096** | 0.64 (NS) | 0.69 | 0.41-1.17 | 0.16 (NS) |  |
| *TATP63* |  |  | -0.006 | 0.95 (NS) | 1.00 (NS) | 0.65 | 0.39-1.11 | 0.11 (NS) |  |
| *TPX2* |  |  | +0.251 | **0.0069** | 0.46 (NS) | 1.52 | 0.90-2.56 | 0.13 (NS) |  |

^a^ Spearman rank correlation Test

^b^ Bonferroni correction

^c^ Hazard ratio (logrank high/low expression level)

^d^ 95% Confidence Interval

^e^ Log-rank test

^f^ Information for 114 patients

*: P <0.05

**: P <0.01

EMT: epithelial-to-mesenchymal transition; OS: overall survival; NS: not significant
