# Supplementary material for: Prognostic value of intratumoral Fusobacterium nucleatum and association with immune-related gene expression in oral squamous cell carcinoma patients
Source: Sci Rep. 2021 Apr 12;11:7870. doi: 10.1038/s41598-021-86816-9 (PMC8041800; doi:10.1038/s41598-021-86816-9)
Supplement: Supplementary file 8 — Supplementary Table S7. [file 41598_2021_86816_MOESM8_ESM.docx]

**Supplementary Table 7. Examples of representative cases showing the relationship between *F. nucleatum* DNA load and lipopolysaccharide (LPS) protein by immunohistochemistry and CD163 mRNA load and immunostaining assessed by Histologic Score (HS).**

| **Samples** | ***F. nucleatum* load**  **(DNA)** | **LPS**  **(Protein)** | **CD163**  **(mRNA)** | **CD163 HS**  **(Protein)** |
| --- | --- | --- | --- | --- |
| **T 634 751** | **709** | **+++** | **0.27** | **+** |
| **T 649 954** | **0.79** | **+/++** | **4.60** | **+/++** |
| **T 616 870** | **0.02** | **+** | **2.13** | **+++** |
| **T 652 415** | **1.30** | **+** | **9.32** | **+++** |
| **T 587 459** | **321** | **+++** | **0.45** | **0/+** |
