# Supplementary material for: Prognostic value of intratumoral Fusobacterium nucleatum and association with immune-related gene expression in oral squamous cell carcinoma patients
Source: Sci Rep. 2021 Apr 12;11:7870. doi: 10.1038/s41598-021-86816-9 (PMC8041800; doi:10.1038/s41598-021-86816-9)
Supplement: Supplementary file 9 — Supplementary Figure S1. [file 41598_2021_86816_MOESM9_ESM.pptx]

## Slide 1
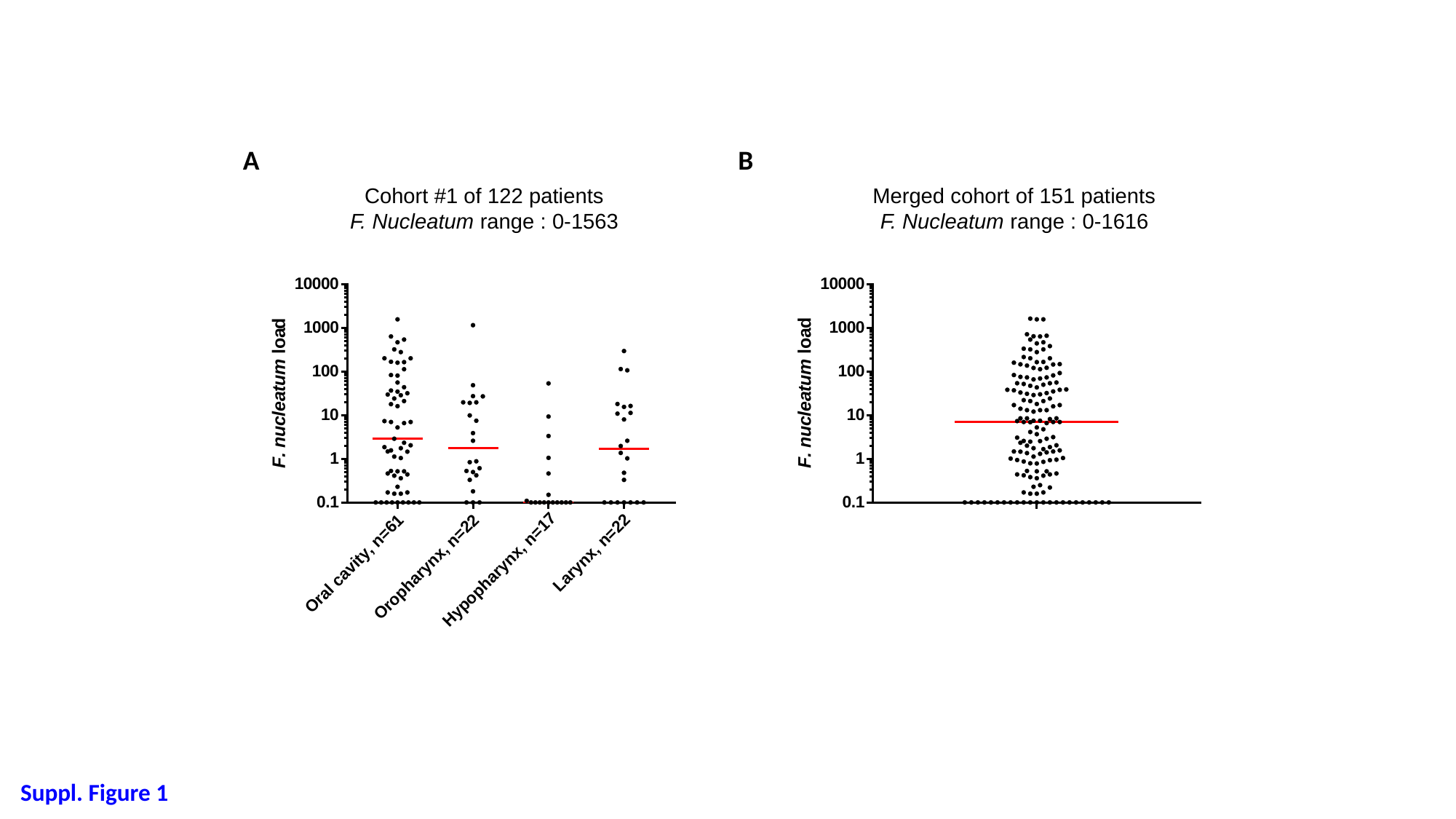

A
B
Cohort #1 of 122 patients
F. Nucleatum range : 0-1563
Merged cohort of 151 patients
F. Nucleatum range : 0-1616
Suppl. Figure 1
