# Supplementary figures and images for: Prognostic value of intratumoral Fusobacterium nucleatum and association with immune-related gene expression in oral squamous cell carcinoma patients
Source: Sci Rep. 2021 Apr 12;11:7870. doi: 10.1038/s41598-021-86816-9 (PMC8041800; doi:10.1038/s41598-021-86816-9)

## Slide 1
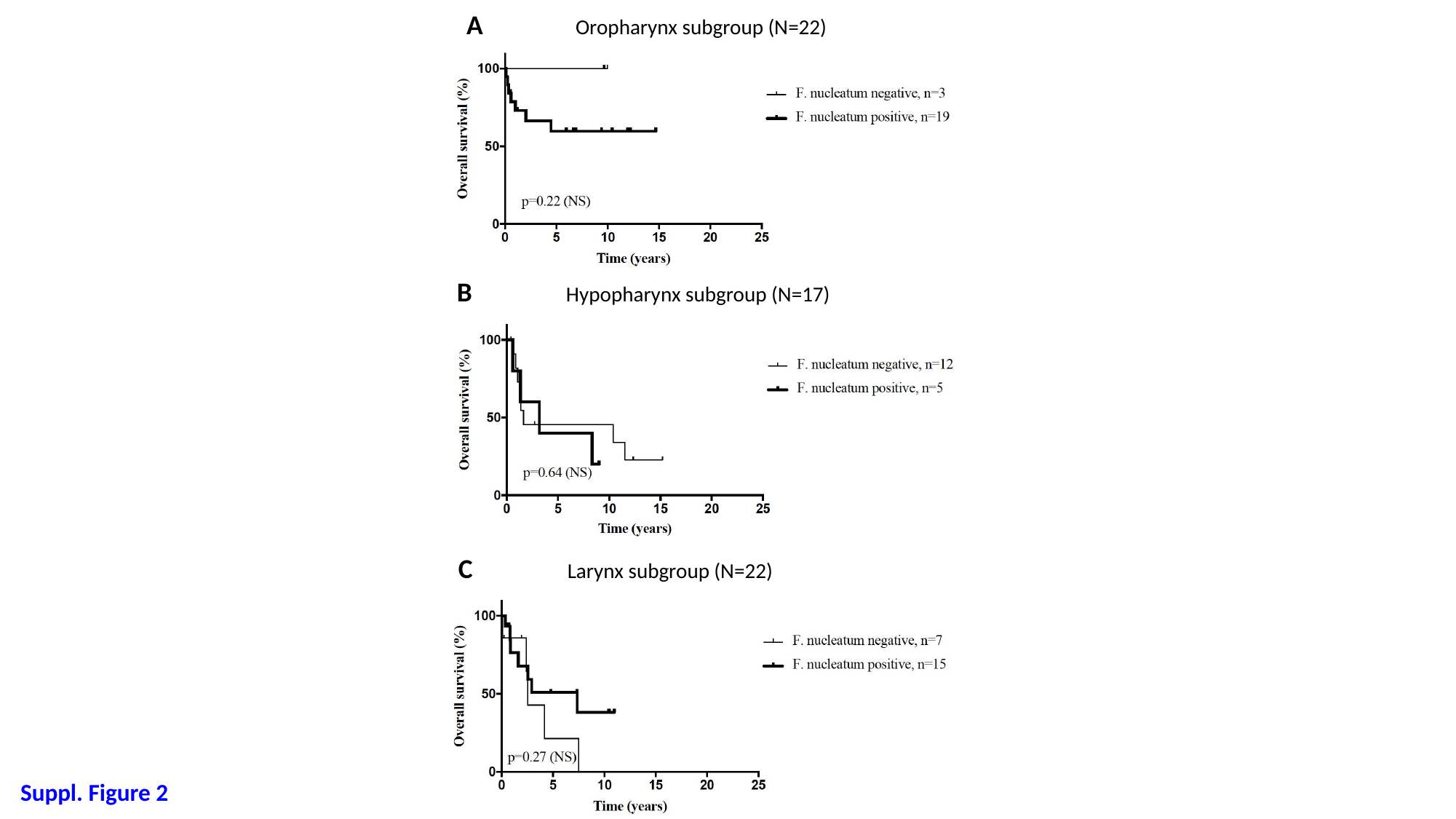

A	Oropharynx subgroup (N=22)
B	Hypopharynx subgroup (N=17)
C	Larynx subgroup (N=22)
Suppl. Figure 2

Supplement: Supplementary file 10 — Supplementary Figure S2. [file 41598_2021_86816_MOESM10_ESM.pptx]

## Slide 1
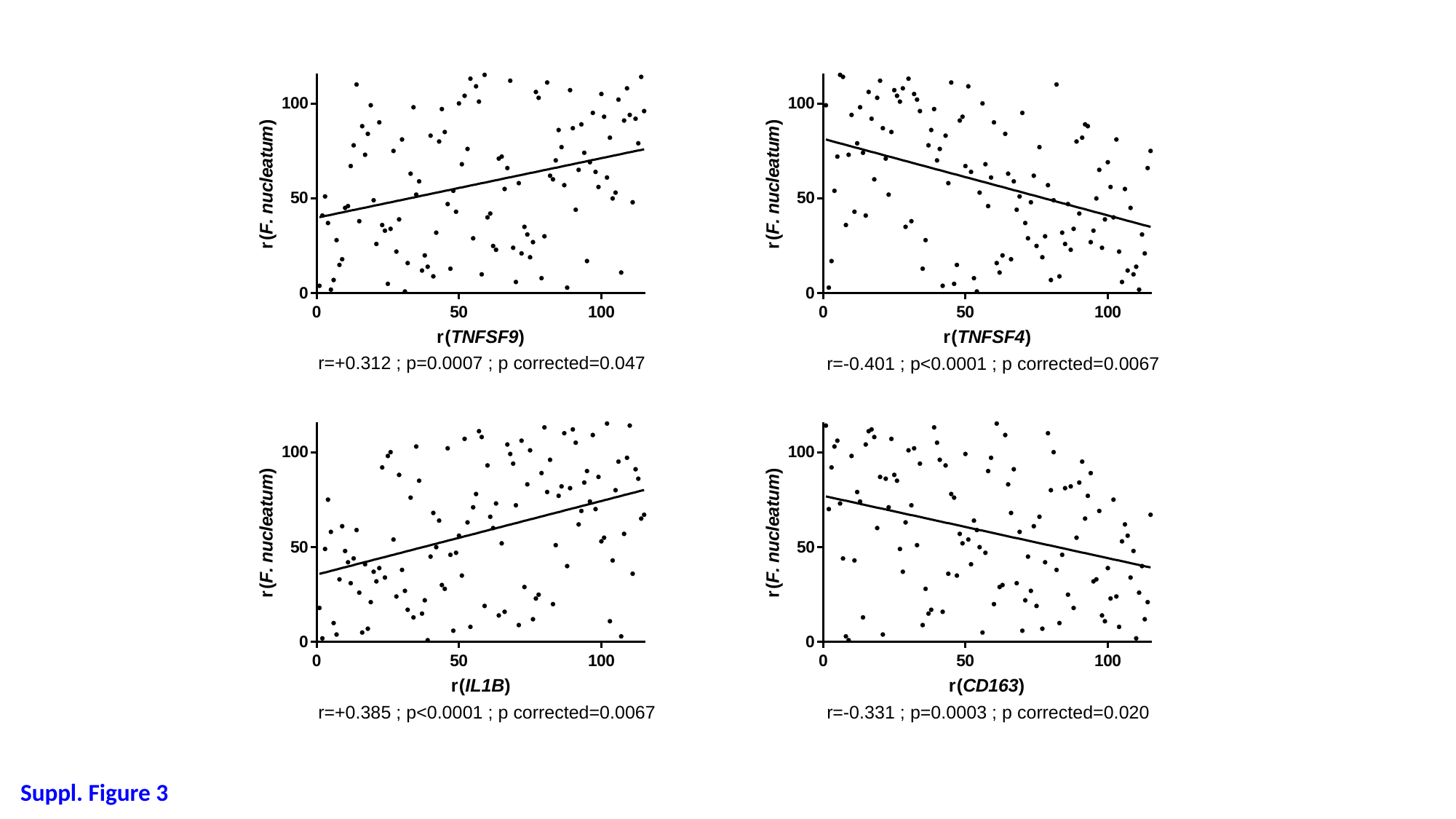

r=+0.312 ; p=0.0007 ; p corrected=0.047
r=-0.401 ; p<0.0001 ; p corrected=0.0067
r=+0.385 ; p<0.0001 ; p corrected=0.0067
r=-0.331 ; p=0.0003 ; p corrected=0.020
Suppl. Figure 3

Supplement: Supplementary file 11 — Supplementary Figure S3. [file 41598_2021_86816_MOESM11_ESM.pptx]
