# Supplementary material for: Prognostic value of intratumoral Fusobacterium nucleatum and association with immune-related gene expression in oral squamous cell carcinoma patients
Source: Sci Rep. 2021 Apr 12;11:7870. doi: 10.1038/s41598-021-86816-9 (PMC8041800; doi:10.1038/s41598-021-86816-9)
Supplement: Supplementary file 12 — Supplementary Figure S4. [file 41598_2021_86816_MOESM12_ESM.pdf]

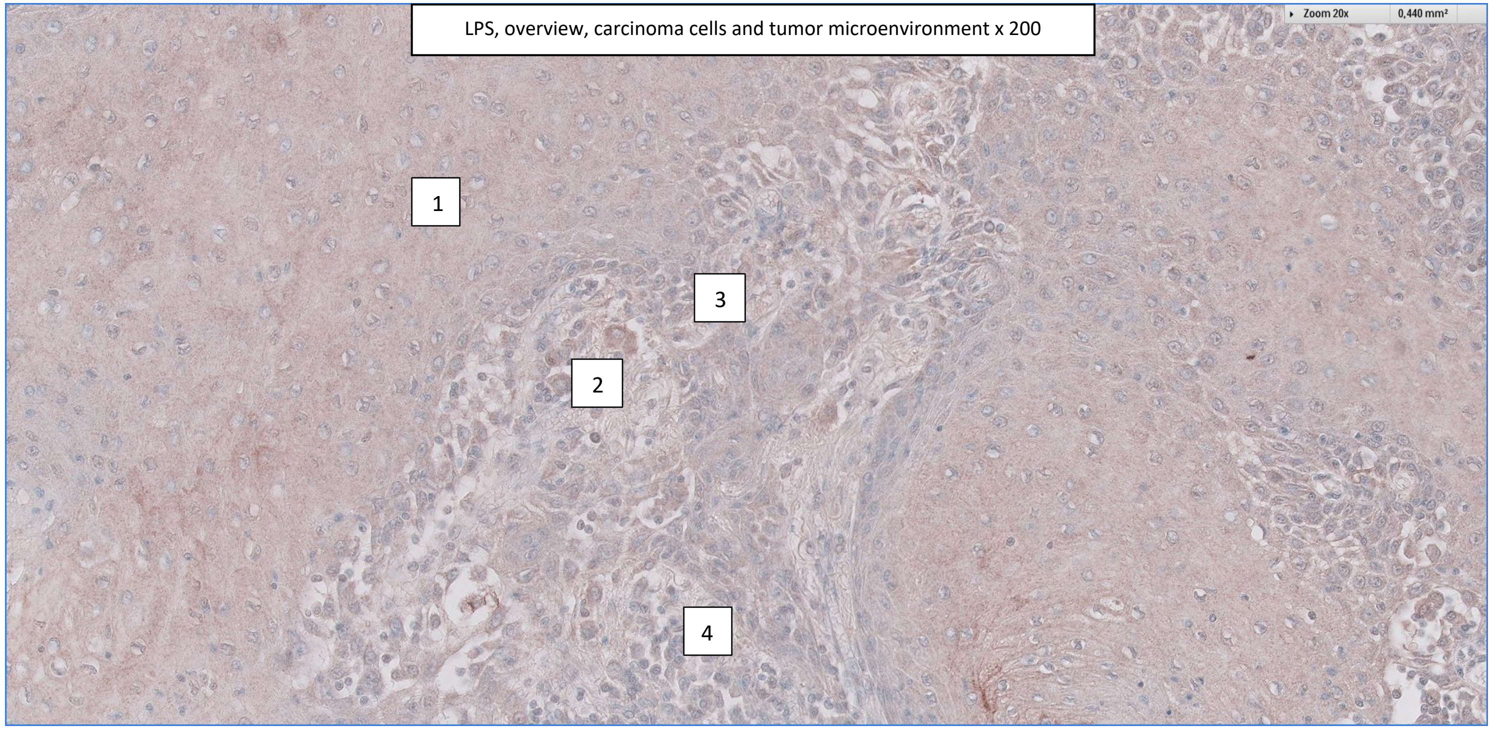

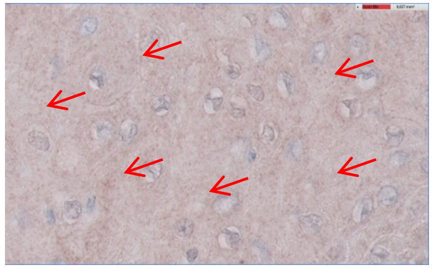

(1) LPS, carcinoma cells, intracellular location x 800

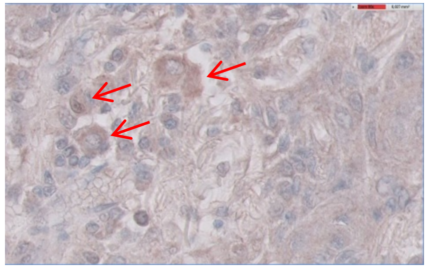

(2) LPS, macrophages, intracellular location x 800

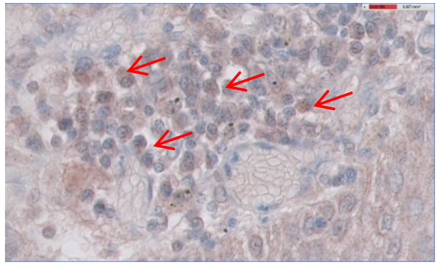

(3) LPS, non-macrophagic immune cells, intracellular location x 800

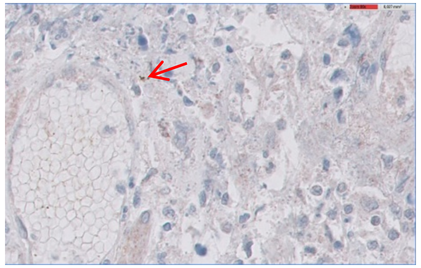

(4) LPS, bacterial extracellular vesicules x 800

Suppl. Figure 4
